# The kinesin motor Klp98A mediates apical to basal Wg transport

**DOI:** 10.1242/dev.186833

**Published:** 2020-08-14

**Authors:** Leonie Witte, Karen Linnemannstöns, Kevin Schmidt, Mona Honemann-Capito, Ferdinand Grawe, Andreas Wodarz, Julia Christina Gross

**Affiliations:** 1 Hematology and Oncology, University Medical Center Goettingen, 37075 Goettingen, Germany; 2 Developmental Biochemistry, University Medical Center Goettingen, 37077 Goettingen, Germany; 3 Molecular Cell Biology, Institute I for Anatomy, University of Cologne Medical School, 50931 Cologne, Germany; 4 Cluster of Excellence-Cellular Stress Response in Aging-Associated Diseases (CECAD), 50931 Cologne, Germany; 5 Center for Molecular Medicine Cologne, University of Cologne, Faculty of Medicine and University Hospital Cologne, 50931 Cologne, Germany

**Keywords:** Kinesin transport, Endosomal trafficking, Transcytosis, Wnt secretion

## Abstract

Development and tissue homeostasis rely on the tight regulation of morphogen secretion. In the *Drosophila* wing imaginal disc epithelium, Wg secretion for long-range signal transduction occurs after apical Wg entry into the endosomal system, followed by secretory endosomal transport. Although Wg release appears to occur from the apical and basal cell sides, its exact post-endocytic fate and the functional relevance of polarized endosomal Wg trafficking are poorly understood. Here, we identify the kinesin-3 family member Klp98A as the master regulator of intracellular Wg transport after apical endocytosis. In the absence of Klp98A, functional mature endosomes accumulate in the apical cytosol, and endosome transport to the basal cytosol is perturbed. Despite the resulting Wg mislocalization, Wg signal transduction occurs normally. We conclude that transcytosis-independent routes for Wg trafficking exist and demonstrate that Wg can be recycled apically via Rab4-recycling endosomes in the absence of Klp98A.

## Abstract (precis)

**Summary:** In the polarized wing disc epithelium of *Drosophila*, Kinesin-like protein 98A mediates transcytosis of multivesicular endosomes.

## INTRODUCTION

During development, formation of a Wnt gradient is necessary for the regulation of cell proliferation and differentiation (Nusse and Clevers, 2017). A prerequisite for the controlled delivery of Wnt from its source to its target cells is the tight regulation of secretory trafficking and the temporal and spatial control of release into the extracellular space. To fine-tune extracellular Wnt levels and to orchestrate long- and short-range Wnt signaling, different routes for Wnt release exist. How Wnt is guided into these respective secretory pathways and the molecular mechanisms involved in directed Wnt trafficking are not yet understood.

Secretory Wnt trafficking can be studied in the polarized *Drosophila* wing imaginal disc epithelium, where Wingless (Wg; *D.mel.* Wnt1) secretion from the dorsoventral boundary, with concentration-dependent activation of signaling, coordinates wing development. After translation and post-translational lipidation at the endoplasmic reticulum (ER), Wg is transported to the apical cell surface together with its cargo receptor, Evi/Wls (Bartscherer et al., 2006). After apical presentation, Wg and Evi enter the endosomal pathway, where Evi is recycled via retromer (Belenkaya et al., 2008; Franch-Marro et al., 2008; Port et al., 2008; Yang et al., 2008). Wg entry into the endosomal pathway has been shown to be necessary for Wg secretion, signal activation and degradation (Pfeiffer et al., 2002). After uptake into the endolysosomal system, Wg-carrying endosomes can be trafficked from the apical towards the basal cell side for transcytosis and basolateral Wg secretion (Yamazaki et al., 2016). Alternatively, Wg can be shuttled back to the apical cell side, where apical Wg secretion and spread could likewise contribute to Wg signal activation (Chang and Sun, 2014; Chaudhary and Boutros, 2019b; Gallet et al., 2008; Linnemannstöns et al., 2020). Whether apical or basal Wg release is the main source of the active Wg signal is still under debate.

After its release, extracellular Wg can activate long-range Wnt signaling over a distance of ≤11 cells (Chaudhary et al., 2019a). The necessity of extracellular Wg spread was highlighted by the finding that a membrane-tethered neurotactin-Wg fusion protein, capable of juxtacrine signal transfer only (Alexandre et al., 2014), cannot induce full Wg signal activation in wing imaginal discs (Chaudhary et al., 2019a) and the adult intestine (Tian et al., 2019). In addition to long-range extracellular spread and short-range transfer to adjacent cells, the Wg signal has been reported to travel on filopodia and cytonemes over a distance of several cell rows (Huang and Kornberg, 2015).

Despite, or perhaps because of, the diversity of the proposed models, our understanding of how secretion and dispersion of the Wnt signal is mediated remains insufficient. So far, the post-endocytic fate of Wg and the molecular mechanisms behind apical and basal Wg secretion are unclear. Tracing how endosomal Wg trafficking for apical or basal secretion is mediated would thus greatly enhance our comprehension of how the Wg signal is transferred.

Here, we analyze the molecular mechanism of apical to basal Wg trafficking by investigating the molecular motors involved in endosomal Wg transport. Molecular motors orchestrate directed organelle transport, and the motor protein-based anterograde and retrograde motility of endosomes governs secretory and degradative processes. Microtubule plus end-directed transport towards the plasma membrane for recycling or secretion is commonly mediated by kinesin motors. Kinesin-3 family members are ideal candidates for transport of endosomal compartments, because they bind to phosphoinositide lipids via their C-terminal PH or PX domains (Lemmon, 2003), and endosomal trafficking processes are tightly regulated by phosphoinositide levels.

We identify kinesin-3 family member Klp98A as the main kinesin responsible for post-endocytic Wg transport. Klp98A is involved in the asymmetric positioning and segregation of Sara signaling endosomes during asymmetric cell division of sensory organ precursors (Derivery et al., 2015). Additionally, Klp98A has previously been shown to mediate autophagosome positioning and fusion in the *Drosophila* fat body by interacting with Atg8 and Rab14 (Mauvezin et al., 2016). Here, we find that Klp98A also mediates apical to basal transport of Wg inside multivesicular endosomes in Wg-producing cells of the wing imaginal disc epithelium. Surprisingly, apical to basal Wg transport by Klp98A is not strictly necessary for Wg signal transduction. We also demonstrate that Wg can be recycled apically via Rab4-recycling endosomes in the absence of Klp98A as an alternative transcytosis-independent way of Wg secretion.

## RESULTS

### Wg mislocalizes to the apical cytosol in the absence of kinesin motor Klp98A

**Fig. 1.**
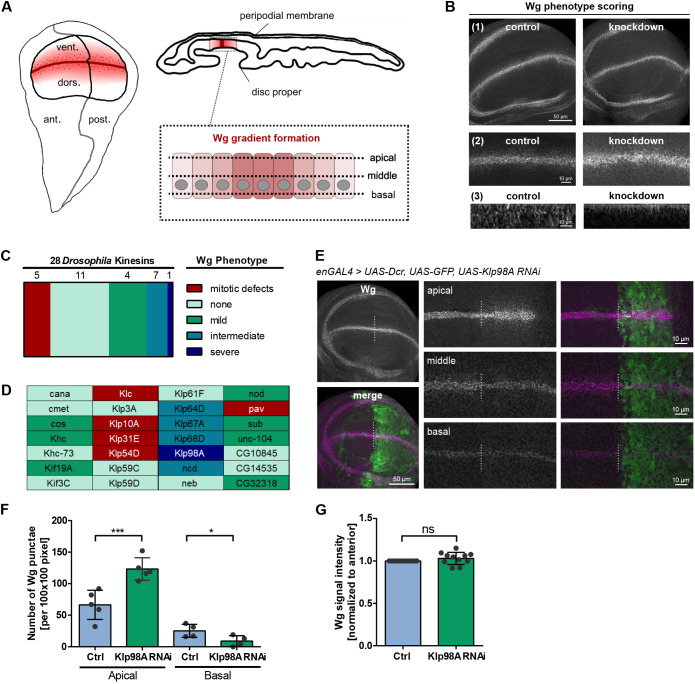
**Wg mislocalizes to the apical cytosol in the absence of kinesin motor Klp98A.** (A) Scheme of the *Drosophila* wing imaginal disc, an epithelial sac composed of an outer peripodial membrane and a columnar epithelial monolayer. Formation of the Wg gradient is mediated by Wg expression along the dorsal-ventral boundary. (B) For RNAi screening, kinesin knockdown was driven by WgGal4, and third instar larval wing imaginal discs were scored for Wg signal distribution within the wing pouch (1), along the wing margin (2) and along the apical-basal axis (3). Cumulative phenotype scores were calculated based on the individual scores in these three categories. (1) and (2) are maximum intensity projections (MIPs); (3) are optical transverse sections of representative control or example knockdown wing imaginal discs. (C,D) WgGal4-driven knockdown of 28 *Drosophila* kinesins induces different degrees of Wg phenotype strength (see also Table S1). (E) EnGal4-driven Klp98A knockdown in posterior wing imaginal discs (marked by co-expression of GFP) induces apical accumulation of Wg in punctate structures while reducing Wg in basal domains. The GFP^−^ compartment serves as an internal control. Overview images (left) are MIPs of apical to basal *z*-stacks, whereas panels show individual confocal sections from apical, intermediate and basal planes of the wing imaginal disc epithelium. Images are representative of three independent experiments with more than six wing imaginal discs. (F) Quantification of punctate Wg in apical or basal sections of anterior and posterior Klp98A knockdown wing imaginal discs. Punctae were quantified individually from the three most apical sections below the peripodial membrane and three basal sections in 100×100 pixel regions of interest. Student's paired two-tailed *t*-test, *P=0.0106, ***P=0.0021. (G) EnGal4-driven Klp98A knockdown does not reduce overall Wg signal intensity in posterior wing imaginal discs. Signal intensity was analysed in 100×100 pixel regions of interest of average intensity projections of anterior and posterior wing imaginal discs separately and normalized to the anterior control. ns, not significant.

To understand the relevance of kinesin-mediated transport of Wg along the microtubule network for Wg secretion, we conducted an *in vivo* RNA interference (RNAi) screen in *Drosophila* wing imaginal discs. The wing imaginal disc is a well-established model system in which to study the Wg secretory pathway. It is an epithelial sac-like structure composed of a polarized epithelium, the disc proper, and an outer squamous cell layer, the peripodial membrane (Fig. 1A). Wg is expressed in a narrow cell population of the polarized wing imaginal disc epithelium along the dorsal/ventral compartment boundary, and Wg secretion from its expression domain via different secretory routes has been implicated in the formation of the Wg gradient (reviewed by Swarup and Verheyen, 2012; Parchure et al., 2018).

To test the function of motor proteins in intracellular Wg transport and Wg secretion, we screened all 28 kinesins found in *Drosophila* (Table S1). Effects of RNAi-mediated knockdown were evaluated based on three aspects: (1) overall Wg protein distribution within the wing pouch; (2) Wg distribution within the expression domain; and (3) intracellular localization along the apical-basal axis within Wg-secreting cells (Fig. 1B). For each category, the arising Wg phenotype was scored from one (comparable to wild type) to three (strong deviation from wild-type Wg distribution) (Fig. S1A,B). Based on this scoring system, a cumulative phenotype score (ranging from three to nine) was calculated to assess the overall relevance of each kinesin for Wg trafficking. Phenotype scores of four and five were regarded as mild and intermediate, respectively, whereas a phenotype score above six was considered as severe. Although the knockdown of 11 kinesins did not affect Wg distribution, several kinesins were found to induce mild (four kinesins) or intermediate (seven kinesins) Wg mislocalization phenotypes (Fig. 1C,D). As expected, this screening approach also returned kinesins involved in mitotic processes, whose knockdown was marked by different degrees of morphological and structural defects (Fig. S1C).

One kinesin, Kinesin-like protein 98A (Klp98A), was found to be most prominently involved in intracellular Wg transport. In RNAi control wing imaginal discs, the Wg signal was distributed throughout the cell from apical towards basal domains, and the majority of Wg was found in distinct punctae (Fig. 1E; Fig. S2A,B). EnGal4-driven knockdown of Klp98A, a member of the kinesin-3 family, led to a significant accumulation of Wg punctae near the apical cell surface (Fig. 1E,F). Concurrently, the amount of intracellular Wg punctae in intermediate and basal domains was reduced. Klp98A knockdown did not affect the overall levels of intracellular Wg signal (Fig. 1G). An intracellular shift and apical accumulation of Wg within distinct punctae were observed irrespective of whether Klp98A knockdown was enGal4 driven in posterior wing imaginal discs or WgGal4 driven in Wg-expressing cells only. EnGal4-driven expression of nontargeting control RNAi did not affect Wg distribution or levels (Fig. S2A,B). Similar phenotypes were observed with three independent Klp98A RNAi lines. From this, we conclude that intracellular transport of Wg involves the kinesin motor Klp98A within Wg-secreting cells.

### Klp98A mediates retrograde transport of endosomal compartments

**Fig. 2.**
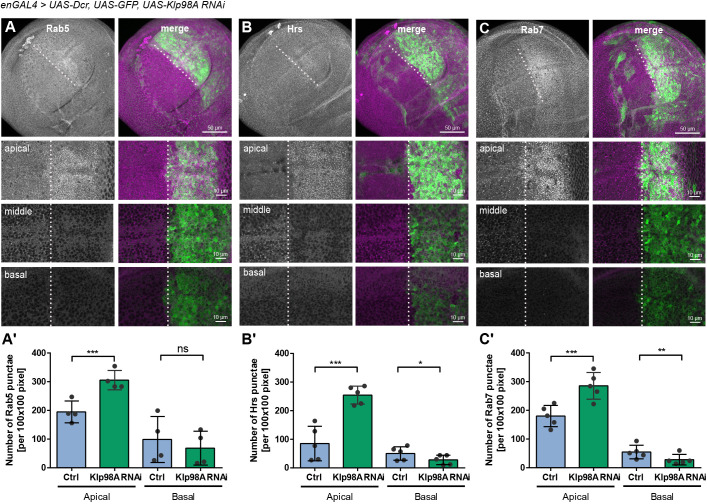
**Klp98A mediates retrograde transport of endosomal compartments.** (A-C) EnGal4-driven Klp98A knockdown in posterior wing imaginal discs (marked by co-expression of GFP) induces apical accumulation and basal reduction of endosomal compartments marked with Rab5 (A), Hrs (B) or Rab7 (C). The GFP^−^ compartment serves as an internal control. Overview images are maximum intensity projections of apical to basal *z*-stacks, whereas panels show individual confocal sections from apical, intermediate and basal planes of the wing imaginal disc epithelium. Images are representative of three independent experiments with more than six wing imaginal discs. (A′-C′) Quantification of Rab5 (A′), Hrs (B′) and Rab7 (C′) punctae in apical and basal sections shows a significant increase in endosomes localizing to the apical cell side and decrease in endosomes localizing to the basal cell side. Endosome punctae were quantified individually from the three most apical sections below the peripodial membrane and three basal sections in 100×100 pixel regions of interest. Data are the mean±s.d. Student's paired two-tailed *t*-test: ***P=0.0004, ns, not significant (A′); *P=0.0107, ***P=0.0003 (B′); **P=0.009, ***P=0.0002 (C′).

In order to understand the functional relevance of Klp98A-mediated transport of Wg, we investigated its role during intracellular Wg transport. Upon translation and post-translational modification, Wg is shuttled from the ER via the Golgi to the apical plasma membrane (Bartscherer et al., 2006; Kadowaki et al., 1996). Klp98A knockdown did not affect the intracellular localization of the ER, marked by calnexin, and the Golgi, marked by GM130 (Fig. S3A,B). Furthermore, DE-cadherin staining was unaffected by Klp98A knockdown (Fig. S3C), indicating that transport from the ER to the plasma membrane does not involve Klp98A-mediated transport. After initial apical presentation, Wg is internalized and shuttled into different proposed secretory routes (Chaudhary and Boutros, 2019b; Gross et al., 2012; Yamazaki et al., 2016) or degraded (Hemalatha et al., 2016). Given that Wg enters the endosomal pathway upon apical endocytosis, the effects of Klp98A knockdown on positioning of the endolysosomal system in wing imaginal discs were analyzed by staining for different early to late endosomal markers. Indeed, enGal4-driven knockdown of Klp98A in posterior wing imaginal discs led to significant apical accumulation of the early endosomal marker Rab5 (Fig. 2A,A′), ESCRT family member Hrs (Fig. 2B,B′) and the late endosomal marker Rab7 (Fig. 2C,C′). Accordingly, the numbers of Rab5^+^, Hrs^+^ and Rab7^+^ endosomes localizing to intermediate and basal domains of the wing imaginal disc were reduced by Klp98A knockdown. EnGal4-driven expression of control RNAi did not affect positioning of endosomal compartments, and no intrinsic anterior-posterior differences were observed (Fig. S2C-F). To check whether Klp98A affects endosomal maturation, we analyzed the colocalization of Rab5, Rab7 and Hrs on endosomal subpopulations in the presence and absence of Klp98A. Given that endosomal maturation occurs by a conversion of Rab5 to Rab7 (Chavrier et al., 1990; Feng et al., 1995), changes in colocalization could be indicative for maturation defects and the generation of hybrid endosomes. Upon Klp98A knockdown, Rab5 colocalization with Rab7 (Fig. S4A,A′) and with Hrs (Fig. S4B,B′) was significantly increased. This increased colocalization of Rab5/Rab7 could stem either from perturbed endosome maturation or from the high density of endosomal structures in the apical cytosol.

### Klp98A knockdown has only a mild effect on endosomal cargo sorting and maturation

**Fig. 3.**
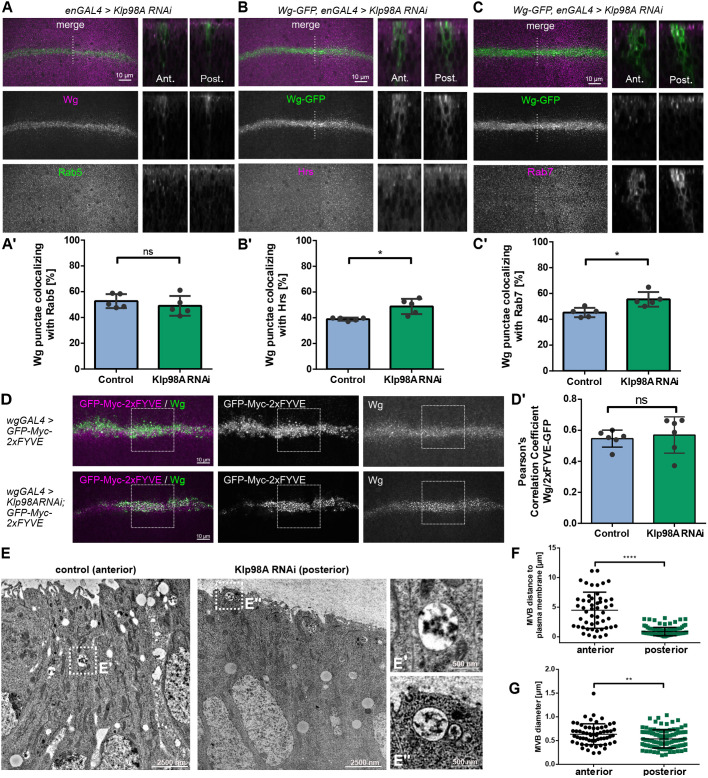
**Klp98A knockdown has only a mild effect on endosomal cargo sorting and maturation.** (A) Colocalization of Wg/Rab5 upon enGal4-driven Klp98A knockdown in posterior wing imaginal discs. Depicted are maximum intensity projections (MIPs) of apical to basal *z*-stacks (left) and optical transverse sections (right). (B,C) Colocalization of endogenously tagged GFP-Wg and Hrs (B) and Rab7 (C) upon enGal4-driven Klp98A knockdown in posterior wing imaginal discs. Depicted are MIPs of apical to basal *z*-stacks (left) and optical transverse sections (right). (A′-C′) The percentage of Wg punctae colocalizing with Rab5 (A′), Hrs (B′) and Rab7 (C′) shows increased colocalization of GFP-Wg with Hrs and Rab7 upon Klp98A knockdown. Images are representative of three independent experiments with more than six wing imaginal discs. Colocalization was analysed in 100×100 pixel regions of interest in the anterior and posterior wing imaginal disc separately. Student's paired two-tailed *t*-test: *P=0.0331 (B′); *P=0.012 (C′). (D) Expression of GFP-myc-FYVE alone or in combination with Klp98A RNAi driven by WgGal4 labels PI(3)P-containing endosomes. MIPs of apical to basal *z*-stacks. (D′) Colocalization of Wg and GFP-myc-FYVE is not affected by Klp98A knockdown. (E,E′,E″) Electron microscopy images of wing imaginal discs with enGal4-driven Klp98A knockdown show apical accumulation of MVE in the posterior Klp98A knockdown compartment. (F) MVE distance to the apical plasma membrane is reduced in cells of the posterior (*n*=117) wing imaginal disc compartment compared with the anterior control (*n*=49). Unpaired Student's two-tailed *t*-test: ****P<0.0001. (G) Quantification of MVE diameter shows a reduction in MVE size between cells of the anterior (*n*=61) and posterior (*n*=106) wing imaginal disc compartment. Unpaired Student's two-tailed *t*-test: **P=0.0029. ns, not significant.

To analyze whether Klp98A knockdown interferes with Wg sorting to endosomal compartments, we analyzed the localization of Wg and endogenous GFP-tagged Wg to endosomal compartments marked with the early to late endosomal markers Rab5, Hrs and Rab7. We observed more pronounced colocalization of Wg and GFP-Wg with Hrs^+^ and Rab7^+^ endosomes upon Klp98A knockdown (Fig. 3A-C,A′-C′). Although this increase in colocalization could stem from slight perturbances in endosomal cargo sorting, it could also be a result of the high density of endosomal structures in the apical cytosol.

To confirm that cargo sorting into endosomes is functional, we used a constitutively active Rab5 mutant (Rab5Q88L). In this mutant, the number of fusion events of endocytic compartments is enhanced, leading to enlarged endosomal compartments that allow visualization of intraluminal content (Zhang et al., 2007). WgGal4-driven expression of Rab5Q88L-YFP led to strongly enlarged endosomal structures that were filled with endogenous Wg and localized mostly to the apical cytosol (Fig. S4C). Similar to control wing imaginal discs, the majority of Wg localized to Rab5Q88L endosomes upon Klp98A knockdown, although endosomes were positioned further towards the apical cytosol and showed a slightly reduced size (Fig. S4C′).

To investigate whether endosomes were still functional in the absence of Klp98A-mediated transport, we analyzed endosomal levels of phosphatidylinositol 3-phosphate [PI(3)P], a crucial factor for recruitment of effector molecules to mature endosomes. Unperturbed PI(3)P levels provide an indirect readout for upstream recruitment of PI(3)-kinases to the apically accumulating endosomes and indicate the possibility for downstream recruitment of endosomal FYVE domain-containing effector proteins. We used 2xFYVE-GFP to mark PI(3)P-containing endosomes (Wucherpfennig et al., 2003). In both control and Klp98A knockdown wing imaginal discs, WgGal4-driven 2xFYVE-GFP colocalized with Wg in punctate structures, corresponding to Wg-carrying PI(3)P-containing endosomes (Fig. 3D). Although PI(3)P-containing endosomes accumulated apically, Klp98A knockdown did not interfere with endosomal PI(3)P recruitment nor did it change colocalization with Wg (Fig. 3D′).

In order to clarify the nature of the observed endosomal compartments, we used electron microscopy (EM). We identified multivesicular endosomes (MVEs) based on their limiting membrane bilayer and the contained intraluminal vesicles. Comparison of MVEs in EM images taken from anterior (control) versus posterior (knockdown) sections of the same wing imaginal disc revealed no morphological defects regarding shape or intraluminal vesicle content. Yet, the knockdown side showed a significantly reduced mean distance of MVEs from the apical plasma membrane and a reduced diameter of 540 nm (knockdown) versus 630 nm (control) (Fig. 3E-G). These data show that Klp98A mediates retrograde transport of MVEs from the plasma membrane towards deeper regions of the cytosol. Although crucial for intracellular MVE positioning, Klp98A is not necessary for general MVE biogenesis, and its knockdown does not negatively interfere with endosomal Wg sorting.

### Klp98A is not necessary for cargo delivery to the lysosome

**Fig. 4.**
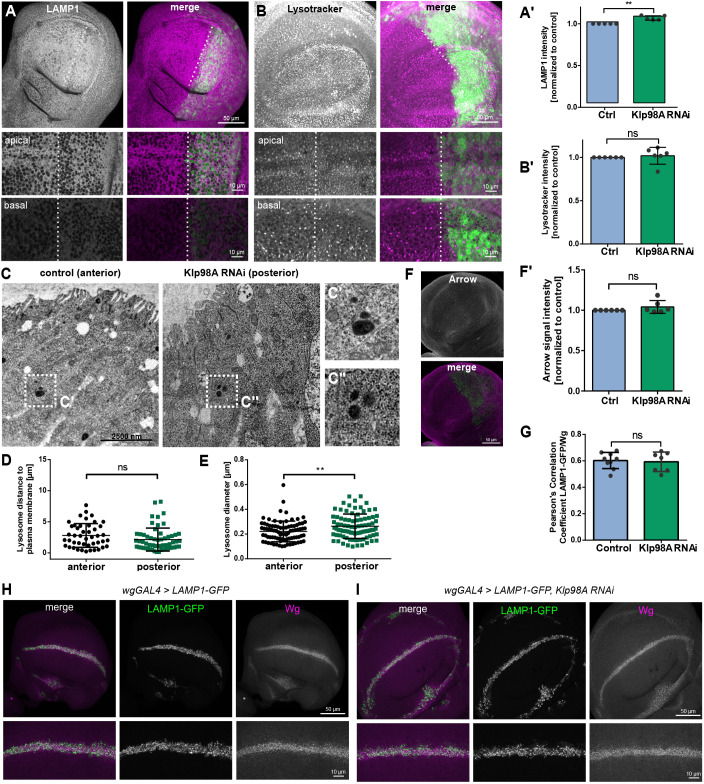
**Klp98A is not necessary for cargo delivery to the lysosome.** (A,B) EnGal4-driven Klp98A knockdown in posterior wing imaginal discs does not affect the localization of lysosomal compartments marked with Lamp1 (A) or Lysotracker staining (B). (A′,B′) Quantification of signal intensities shows increased intensity of Lamp1 (A′) but not Lysotracker staining (B′) upon Klp98A knockdown. Signal intensity was analysed in 100×100 pixel regions of interest of average intensity projections of anterior and posterior wing imaginal discs separately and normalized to the anterior control. Student's paired two-tailed *t*-test: **P=0.0043. (C-C″) Electron microscopy images of wing imaginal discs with enGal4-driven Klp98A knockdown show normal lysosome positioning in the posterior Klp98A knockdown compartment. (D) Lysosome distance to the apical plasma membrane is unchanged in cells of the posterior (*n*=86) wing imaginal disc compartment compared with the anterior control (*n*=86). (E) Quantification of lysosome diameter shows an increase in lysosome size between the anterior (*n*=86) and posterior (*n*=86) wing imaginal disc compartment. Unpaired Student's two-tailed *t*-test: **P=0.003. (F,F′) EnGal4-driven Klp98A knockdown in posterior wing imaginal discs does not lead to accumulation of Arrow. Intermediate section (F) and quantification of signal intensities (F′). (G-I) Expression of GFP-Lamp1 alone (G) or in combination with Klp98A RNAi (H) driven by WgGal4 shows that colocalization of Wg and GFP-Lamp1 is not affected by Klp98A knockdown. Pearson's correlation coefficient (G) and maximum intensity projections of apical to basal *z*-stacks (H,I). ns, not significant.

In general, MVEs are guided into one of two major trafficking pathways: towards the lysosome for degradation or towards the plasma membrane for fusion and release of intraluminal vesicles (Palmulli and Van Niel, 2018). To investigate whether Klp98A transports endosomes towards degradation, we analyzed lysosome positioning and acidification upon Klp98A knockdown. Positioning of Lamp1^+^ lysosomal compartments was unaffected by Klp98A RNAi, although a slight increase in Lamp1 signal intensity was observed (Fig. 4A,A′). Likewise, Klp98A knockdown affected neither the intensity nor the positioning of acidic lysosomal compartments marked with Lysotracker staining (Fig. 4B,B′). Electron microscopy confirmed normal lysosome positioning, with lysosomes localizing to a similar distance from the plasma membrane in control and Klp98A knockdown compartments of wing imaginal discs (Fig. 4C,D). The diameter of lysosomes was increased by Klp98A knockdown (Fig. 4E).

Given that defects in lysosomal degradation by knockdown of the endosomal component Hrs induce intracellular accumulation of Wg and its receptors (Rives et al., 2006), we next investigated the role of Klp98A specifically in the degradation of Wg and its receptor, Arrow. As observed for Wg (Fig. 1E,G), Arrow did not accumulate upon Klp98A knockdown (Fig. 4F,F′). Furthermore, colocalization of Lamp1-GFP with Wg was similar in both control and Klp98A knockdown compartments (Fig. 4G-I). These data indicate that cargo transport to lysosomal compartments does not involve Klp98A. Taken together, we conclude that Klp98A does not play a role in lysosomal transport, acidification and degradation.

### Klp98A mediates apical to basal Wg transcytosis

**Fig. 5.**
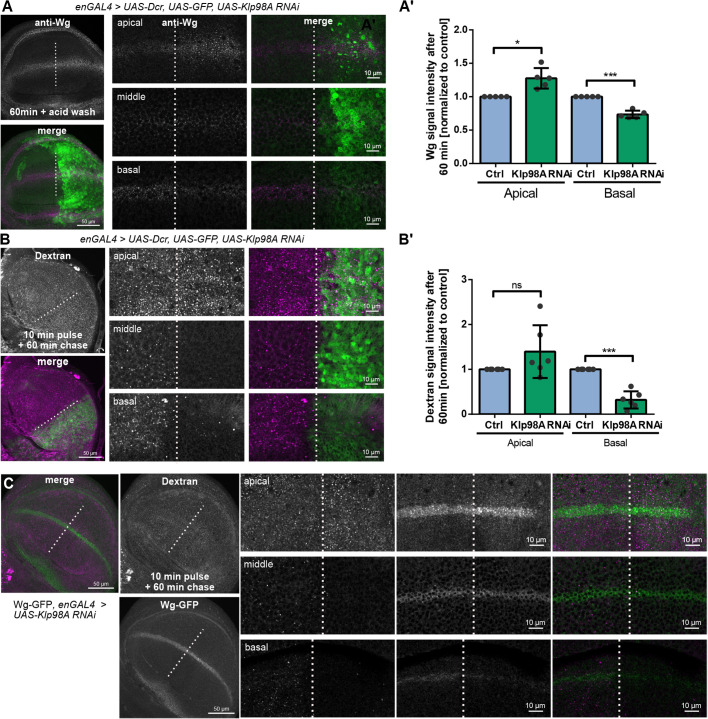
**Klp98A mediates apical to basal Wg transport.** (A) Endocytosis of anti-Wg antibody for 60 min in enGal4-driven Klp98A knockdown wing imaginal discs. Overview images are maximum intensity projections (MIPs) of apical to basal *z*-stacks, whereas panels show individual confocal sections from apical, intermediate and basal planes of the wing imaginal disc epithelium. (A′) Quantification of Wg signal intensity in apical versus basal confocal sections shows apical accumulation and basal reduction of post-endocytic Wg signal 60 min after endocytosis upon Klp98A knockdown. The average Wg signal intensity in the anterior and posterior wing imaginal disc of 150×150 pixel regions of interest of three apical and three basal sections was quantified. The posterior knockdown compartment was normalized to the anterior control. Student's paired two-tailed *t*-test: *P=0.016; ***P=0.0004. (B) Endocytosis of Dextran-A568 after 10 min pulse and 60 min chase in enGal4-driven Klp98A knockdown wing imaginal discs. Overview images are MIPs of apical to basal *z*-stacks, whereas panels show individual confocal sections from apical, intermediate and basal planes of the wing imaginal disc epithelium. (B′) Quantification of Dextran signal intensity in apical versus basal confocal sections shows apical accumulation and basal reduction of Dextran signal 60 min after endocytosis upon Klp98A knockdown. The average Dextran signal intensity in the anterior and posterior wing imaginal discs of 150×150 pixel regions of interest of three apical and three basal sections was quantified. The posterior knockdown compartment was normalized to the anterior control. Student's paired two-tailed *t*-test: ***P=0.003. ns, not significant. (C) Endocytosis of Dextran-A568 for 60 min after 10 min pulse in enGal4-driven Klp98A knockdown wing imaginal discs expressing endogenously tagged Wg-GFP. Overview images are MIPs of apical to basal *z*-stacks, whereas panels show individual confocal sections from apical, intermediate and basal planes of the wing imaginal disc epithelium. Images are representative of three independent experiments with more than six wing imaginal discs.

Wg secretion for long-range signal transduction has been proposed to occur after apical presentation and transcytosis to the basal plasma membrane (Gallet et al., 2008; Yamazaki et al., 2016). To investigate the role of Klp98A for apical to basal Wg trafficking, we monitored endocytosis and intracellular trafficking of Wg after surface labeling with monoclonal anti-Wg antibody (Hemalatha et al., 2016). To exclude signal stemming from surface-bound or extracellular Wg and to visualize intracellular endosomal Wg trafficking exclusively downstream of apical presentation, extracellularly bound antibody was removed by acid washing. In control compartments, 60 min after antibody addition, surface-labeled Wg localized mostly to the basal cytosol, with distinct intracellular Wg punctae in intermediate sections (Fig. 5A). In the posterior Klp98A knockdown compartment, however, intracellular Wg localized predominantly to punctate apical structures. This apical Wg accumulation was accompanied by a strong reduction of intermediate and basal Wg signal and an apparent abrogation of apical to basal intracellular Wg shuttling (Fig. 5A′). To understand where endosomal Wg is targeted after apical endocytosis, we next analyzed the colocalization of endocytosed Wg and Lamp1-GFP. Sixty minutes after antibody addition, the overall colocalization of endocytosed Wg with Lamp1-GFP was low, indicating that Wg is not targeted mainly for lysosomal degradation upon apical entry into the endosomal system (Fig. S5A). In line with our previous findings, Klp98A knockdown did not visibly affect the colocalization of Lamp1-GFP and Wg. Taken together, we conclude that upon apical endocytosis, Wg is transcytosed to the basal membrane in a Klp98A-dependent manner.

To investigate the role of Klp98A in transcytosis further, endocytosis of Dextran was monitored after 10 or 60 min of incubation with fluorochrome-labeled Dextran. Wing imaginal discs fixed after a 10 min pulse showed Dextran^+^ vesicles in both the apical and the basal cytosol of control and knockdown compartments of wing imaginal discs (Fig. S5B). This indicates that Klp98A knockdown does not interfere with apical or basal endocytosis per se. Additionally*,* in control but not in Klp98A knockdown compartments, several Dextran particles were detected in intermediate cell regions, showing that endocytic vesicles move from the surface into deeper cell regions within a few minutes. After 60 min, Dextran-carrying endosomes localized to the apical and basal cytosol in the control compartment, and Dextran^+^ vesicles were additionally present in deeper cell sections (Fig. 5B). In the absence of Klp98A, Dextran-marked endocytic vesicles accumulated apically, and basal Dextran signal intensity was significantly reduced (Fig. 5B′), thus mimicking the effects observed for surface-labeled Wg. Colocalization of Dextran with GFP-Wg provided further confirmation that Dextran and Wg are trafficked within the same endosomal compartments after endocytosis (Fig. 5C). These findings confirm that endocytic cargo, such as Wg and Dextran, is trafficked towards the basal cytosol within 60 min after apical endocytosis in control conditions, but not in the absence of Klp98A. We conclude that the kinesin motor Klp98A mediates retrograde transport of endosomal compartments from the apical to the basal wing imaginal disc cytosol. Furthermore, we showed that upon apical entry into the endosomal system, Wg is shuttled towards the basal cytosol and that apical to basal Wg trafficking depends on Klp98A-mediated endosome transport.

Given that signaling-competent Wg was previously described to originate from the basal side of the wing imaginal disc epithelium, these findings raise the question of whether Klp98A-mediated apical to basal transcytosis is necessary for Wg signal transduction.

### Apical to basal Wg transcytosis via Klp98A is not strictly essential for Wg signal propagation

**Fig. 6.**
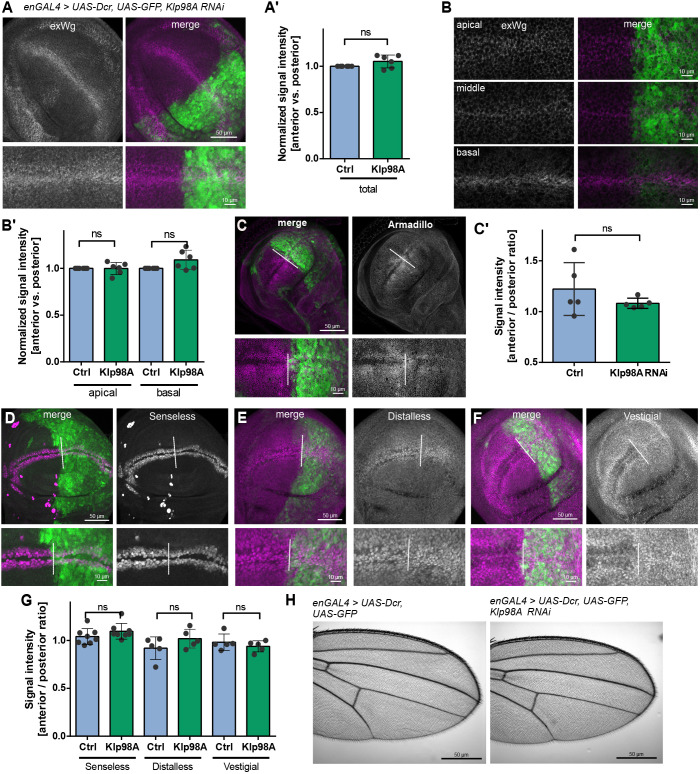
**Apical to basal Wg transcytosis via Klp98A is not strictly essential for Wg signal propagation.** (A,B) EnGal4-driven Klp98A knockdown in posterior wing imaginal discs does not reduce extracellular Wg levels. Depicted are maximum intensity projections (MIPs) (A) and individual confocal sections from apical, intermediate and basal planes of the wing imaginal disc epithelium (B). (A′,B′) Quantification of extracellular Wg signal intensity shows no change in overall (A′) and in apical or basal (B′) extracellular Wg intensity upon Klp98A knockdown. The average extracellular Wg signal intensity in the anterior and posterior wing imaginal disc of 250×250 pixel regions of interest of five apical and five basal sections and of the whole apical to basal *z*-stack was quantified individually. The posterior knockdown compartment was normalized to the anterior control. (C) EnGal4-driven Klp98A knockdown does not affect localization or stability of Arm. Depicted are MIPs of apical to basal *z*-stacks. Images are representative of three independent experiments with more than six wing imaginal discs. (C′) Quantification of signal intensity shows no increase in Arm levels. Signal intensity was analysed in 100×100 pixel regions of interest of average intensity projections of anterior and posterior wing imaginal discs separately and normalized to the anterior control. (D-F) EnGal4-driven Klp98A knockdown does not affect the stability of Wg targets Senseless (D), Distalless (E) and Vestigial (F). Depicted are MIPs of apical to basal *z*-stacks. Images are representative of three independent experiments with more than six wing imaginal discs. (G) Ratios of anterior versus posterior signal intensities of control and enGal4-driven Klp98A knockdown wing imaginal discs show no difference in anterior/posterior signal intensity upon Klp98A knockdown. Student's two-tailed *t*-test: *P*=0.2013 (Senseless), *P*=0.1884 (Distalless), *P*=0.3774 (Vestigial). Two one-sided hypothesis testing confirmed statistical equivalence with an indifference threshold of 0.25 (95% confidence). (H) EnGal4-driven Klp98A knockdown does not induce wing notches. ns, not significant.

To understand whether the strong apical mislocalization of Wg-containing endosomes and the inhibition of apical to basal Wg transport affect Wg signal transfer, we investigated Wg secretion and signal transduction upon Klp98A knockdown. Staining for extracellular Wg in nonpermeabilized wing imaginal discs revealed that, surprisingly, Klp98A knockdown did not affect apical or basolateral extracellular Wg levels. Likewise, the overall intensity of extracellular Wg staining was not reduced (Fig. 6A-B′). Extracellular staining of endogenous GFP-tagged Wg validated these findings, because apical and basal extracellular GFP levels were unchanged upon Klp98A knockdown (Fig. S6A), indicating that Klp98A-mediated apical to basal transport of Wg is not necessary for extracellular Wg levels.

To investigate the role of Klp98A-mediated Wg transport for Wg signal transduction, we examined Armadillo (Arm; *D.mel.* beta-catenin) and the levels of several proteins encoded by Wnt target genes. Given that activation of Wg signaling leads to stabilization of cytoplasmatic Arm, interference with Wg signaling affects cytoplasmic Arm protein levels (Kim et al., 2016; Wu and Nusse, 2002). Staining for Arm did not show alterations between control and Klp98A knockdown wing imaginal discs regarding the formation of the characteristic stabilization pattern (Fig. 6C) and the overall signal intensity (Fig. 6C′). Likewise, enGal4-mediated Klp98A knockdown did not affect expression of the short-range Wg target Senseless (Fig. 6D) and of the long-range Wg targets Distalless and Vestigial (Fig. 6E,F). For quantitative analysis of Wg target gene activation, ratios of anterior versus posterior signal intensities of control and enGal4-driven Klp98A knockdown wing imaginal discs were compared, because the respective staining patterns show intrinsic anterior versus posterior differences. Knockdown of Klp98A in the posterior wing imaginal disc compartment did not affect the ratio of anterior versus posterior expression of all investigated Wg targets (Fig. 6G). With comparable staining and sample sizes, other genes involved in Wnt signaling, such as *Sec6* (Chaudhary and Boutros, 2019b), previously showed an effect on Wnt target gene expression. True equivalency was confirmed by statistical equivalence testing. In line with these findings, knockdown of Klp98A in the posterior compartment (enGal4) or in Wg-expressing cells (WgGal4) and using different RNAi lines did not induce wing notches in the adult fly (Fig. 6H; Fig. S6B,C). Taken together, these findings strongly indicate that Klp98A-mediated endosomal transport, although necessary for intracellular Wg trafficking to perinuclear and basal domains, is not strictly essential for Wg gradient formation and signal transduction.

### Klp98A-independent Wg recycling is sufficient for signal propagation

**Fig. 7.**
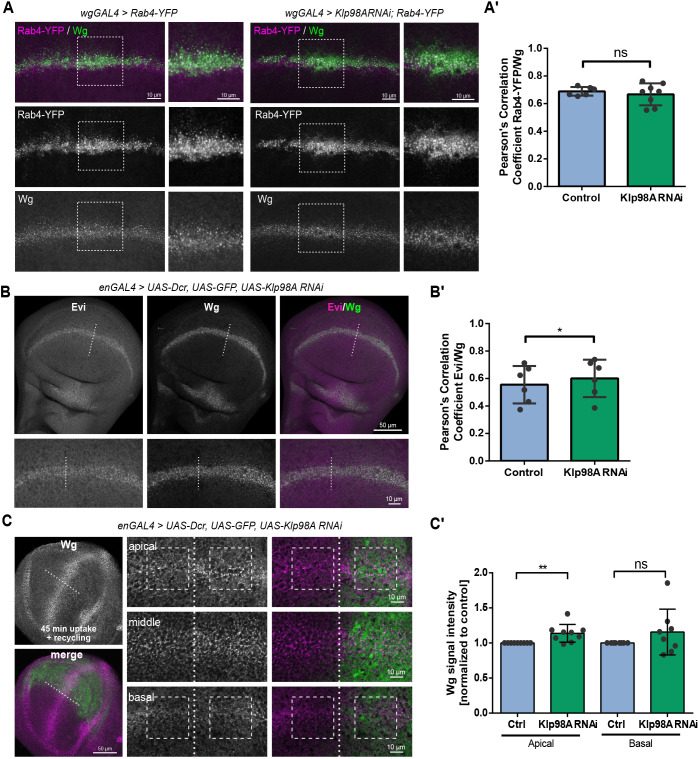
**Klp98A-independent Wg recycling is sufficient for signal propagation.** (A) Expression of Rab4-YFP alone or in combination with Klp98A RNAi driven by WgGal4 labels Rab4-recycling endosomes. Maximum intensity projections (MIPs) of apical to basal *z*-stacks. (A′) Colocalization of Wg and Rab4-YFP is not affected by Klp98A knockdown. (B) Colocalization of Wg and Evi is slightly increased upon enGal4-driven Klp98A knockdown in posterior wing imaginal discs. Depicted are MIPs of apical to basal *z*-stacks. (B′) Pearson correlation coefficient of Wg/Evi is increased upon Klp98A knockdown. Colocalization was analysed in 100×100 pixel regions of interest in the anterior and posterior wing imaginal discs separately and the posterior knockdown compartment normalized to the anterior control. Images are representative of three independent experiments with more than six wing imaginal discs. Student's paired two-tailed *t*-test: *P=0.0177. (C) Live staining with anti-Wg antibody for 45 min in enGal4-driven Klp98A knockdown wing imaginal discs shows increased apical Wg secretion. Overview images are MIPs of apical to basal *z*-stacks, whereas panels show individual confocal sections from apical, intermediate and basal planes of the wing imaginal disc epithelium. Images are representative of three independent experiments with more than six wing imaginal discs. Boxes indicate exemplary regions of interest of 150×150 pixels used for quantification. (C′) Quantification of Wg signal intensity in apical versus basal confocal sections shows increased apical Wg secretion upon Klp98A knockdown. The average Wg signal intensity in the anterior and posterior wing imaginal disc of 150×150 pixel regions of interest of three apical and three basal sections of eight wing imaginal discs from three independent experiments was quantified. The posterior knockdown compartment was normalized to the anterior control. Student's paired two-tailed *t*-test: **P=0.0117. ns, not significant.

Proper Wg secretion for signal transduction and wing development in the absence of Klp98A could be explained by transcytosis-independent routes for Wg secretion. Previous studies have proposed multiple models for Wg secretion from endosomal compartments, including the release on extracellular vesicles, cytoneme-mediated transport and transcytosis followed by basolateral secretion (Gross et al., 2012; Huang and Kornberg, 2015; Yamazaki et al., 2016). Additionally, Rab4-mediated recycling from early endosomes has been suggested to allow fast retrieval and apical re-secretion of the lipid-modified morphogen Hedgehog in wing imaginal discs (D'Angelo et al., 2015), and a similar model has been proposed for Wg (Gao et al., 2017; Linnemannstöns et al., 2020). In control wing imaginal discs, a substantial proportion of Wg colocalized with Rab4-YFP, mostly in apical vesicular structures (Fig. 7A). Upon Klp98A knockdown, although Rab4^+^ compartments localized even more apically, the proportion of Wg co-localizing with Rab4 was unchanged (Fig. 7A′). This indicates that Rab4-mediated recycling of Wg can occur in the absence of Klp98A-mediated endosomal transport and fits with the idea that Rab4-recycling occurs from apical compartments after endocytosis.

If Klp98A-mediated endosomal transport is not necessary for Wnt secretion, we reasoned that the Wg cargo receptor, Evi, whose endosomal retrieval after endocytosis is necessary for Wg secretion, should not be affected by Klp98A knockdown (Franch-Marro et al., 2008; Pradhan-Sundd and Verheyen, 2014). As expected, Klp98A knockdown had no effect on Evi localization or stability in wing imaginal disc cells (Fig. 7B) and did not reduce colocalization of Wg and Evi within recycling endosomes (Fig. 7B′). Consequently, Evi recycling from early endosomes does not depend on Klp98A. This supports the idea that Wg and Evi are separated in endosomal compartments early after endocytosis, from where Evi is quickly retrieved via Retromer, whereas Wg is recycled back to the plasma membrane via Rab4-recycling endosomes.

In order to visualize apical Wg secretion upon Klp98A knockdown, a nonpermeabilizing live staining protocol was performed, for which wing imaginal discs were incubated with anti-Wg antibody for 45 min at 22°C. As opposed to conventional extracellular Wg staining at 4°C, endocytosis and secretion events persist at this permissive temperature, and secreted Wg can be antibody captured on the wing imaginal disc surface. Upon enGal4-driven Klp98A knockdown, a slight but significant increase in apical Wg signal was observed (Fig. 7C,C′). This indicates that Klp98A knockdown enhanced rather than interfered with apical Wg recycling, an effect that is likely to result from the close proximity of endosomal compartments to the apical cell side that could facilitate apical Rab4 recycling. Similar to conventional extracellular Wg staining, Klp98A knockdown did not reduce basal Wg signals at 22°C, suggesting that basal extracellular Wg levels might not depend on basal endosomal secretion.

With these data, we show that Wg can be recycled apically via Rab4^+^-recycling endosomes and that this recycling route is independent of Klp98A-mediated endosomal transport. Rab4-mediated apical Wg secretion therefore provides one possible mechanism of apical Wg spreading and could explain how normal Wg signal transduction takes place in the absence of Klp98A-mediated Wg transcytosis.

## DISCUSSION

Long-range Wg transfer depends on Wg entry into the endosomal system followed by secretory endosomal transport. In the polarized epithelium of *Drosophila* wing imaginal discs, many different intracellular trafficking routes for endosomal Wg have been discovered, yet the molecular mechanisms involved have remained poorly understood. Here, we find that upon apical endocytosis, Wg is transported towards basal cellular domains by the kinesin motor Klp98A. Surprisingly, apical to basal transport of endosomal Wg via Klp98A is dispensable for Wg signal transduction, and blocking Wg transcytosis by Klp98A knockdown does not interfere with extracellular levels of Wg, subsequent target gene activation and wing development. Normal long- and short-range signal transduction in the absence of Klp98A-mediated apical to basal Wg transport indicates that basal endosomal Wg might not be necessary for Wg signal transduction. It is thus conceivable that transcytosis-independent routes for Wg secretion and extracellular spread are necessary and sufficient for Wg signal activation. Klp98A-independent signal transfer could, however, also be attributable to possible redundancy with other kinesins or an early gradient of Wg expression in this tissue that could bypass the need for secretion and long-range spread of Wg (Alexandre et al., 2014).

We provide evidence that upon apical endocytosis, Wg is shuttled into Rab4^+^-recycling endosomes independently of Klp98A and thus propose that apical Wg secretion from Rab4-recycling endosomes is sufficient to maintain normal extracellular Wg levels. Our finding that apical recycling of Wg could be the major contributor to Wg signal activation is surprising, because apical to basal Wg transport was thought to feed long-range Wg secretion by shuttling Wg into its reported secretory pathways (Gallet et al., 2008; Yamazaki et al., 2016). In agreement with our data, analysis of the timecourse of Wg trafficking has revealed that post-endocytic Wg is transported from the apical towards the basal cytosol (Yamazaki et al., 2016). Interestingly, knockdown of the E3-ligase Godzilla was found to impair both Wg signal transduction and apical to basal Wg transport, strengthening the idea of basolateral signal secretion (Yamazaki et al., 2016). Our data indicate that basal endosomal Wg might, however, not be necessary for Wg signal activation and lead to the conclusion that basal endosomal secretion, if it occurs, does not make a major contribution to signal activation. This finding implies additional functions for Godzilla during post-endocytic Wg trafficking and requires the careful review of previous models for long-range Wg spreading.

Given that our data call into question the reason why Klp98A-mediated Wg transport towards the basal cell side takes place, we carefully investigated whether Klp98A is involved in Wg degradation. In Wg-receiving cells, degradation restricts Wg extracellular spread and shapes gradient formation (Marois et al., 2006; Piddini et al., 2005; Strigini and Cohen, 2000), but it is not clear whether lysosomal targeting of Wg also plays a role in Wg-secreting cells. We found that Klp98A-mediated retrograde Wg transport is not involved in degradative endosomal trafficking and instead shuttles Wg towards the basolateral cell side. Interestingly, although intracellular basal Wg levels were dramatically reduced upon Klp98A knockdown, no such polarity was observed for extracellular Wg. Regardless of whether transcytosis was blocked, extracellular Wg localized to the basal cell surface. This suggests that the extracellular organization of Wg distribution does not arise from polarized secondary secretion at the basal cell surface. Instead, Wg attachment to cell-surface heparane sulfate proteoglycans (HSPGs) or its receptors could determine the extracellular localization of Wg (Baeg et al., 2001), although direct post-Golgi routes towards the basal plasma membrane are also conceivable. Glypicans, such as Dally-like, have been reported to facilitate apical Wg internalization and transport to the basal membrane, where they could accumulate together with their cargo (Gallet et al., 2008). In addition to HSPGs, Wg receptor Dfz2 also predominantly localizes to the basal epithelium surface, from where it is endocytosed for signal initiation (Hemalatha et al., 2016). Interestingly, the *Drosophila* metalloproteinase Sol Narae (Sona) localizes to the basal extracellular matrix of the wing epithelium, where it cleaves extracellular Wg (Kim et al., 2016; Won et al., 2019). The resulting Wg-C-terminal fragment induces a discrete subset of Wg response genes to regulate cell proliferation specifically (Won et al., 2019). Therefore, basal extracellular Wg could fuel the generation of a C-terminal Wg-fragment and thereby control cell proliferation during wing imaginal disc development. Basal extracellular Wg or a C-terminal Wg-fragment could also serve as a ligand pool for target cell cytonemes to pick up Wg ligands (Huang and Kornberg, 2015).

Based on our data, neither organization of the extracellular Wg signal nor Wg signal transfer depends on basal Wg release from endosomal organelles. Where does the Wg signal come from? Based on our findings, we propose that Wg, similar to Hedgehog (Hh), is recycled apically via Rab4-recycling endosomes. Owing to their shared lipid modifications and resulting hydrophobicity, both morphogens show similar trafficking behaviour, leading to similar intracellular trafficking routes. Similar to Wg, Hh undergoes apical presentation and endocytic reuptake before the signal is finally released (Gradilla et al., 2018; Parchure et al., 2018). Subsequent Hh signal transduction can mainly occur by two mechanisms: (1) basolateral secretion on extracellular vesicles (Bischoff et al., 2013; Gradilla et al., 2014); and (2) apical recycling via Rab4 endosomes (D'Angelo et al., 2015). The extracellular vesicles represent an established route of long-range Wg spread (Greco et al., 2001; Gross et al., 2012), whereas apical recycling via Rab4 endosomes has not been considered necessary for Wg signal transduction, although Wg colocalization with Rab4 has been reported previously (Gao et al., 2017; Linnemannstöns et al., 2020). Apical Wg secretion is supported by previous reports of Wg being found on the apical surface of receiving cells at a distance of several rows from its source (Gallet et al., 2008). A recent study also confirmed that interference with exocyst-mediated apical Wg secretion leads to reduced downstream signal activation and defects in wing development (Chaudhary and Boutros, 2019b). Moreover, apical or lateral Rab4-mediated Wg recycling aligns well with other described mechanisms of Wg spread, because it does not contradict Wg packaging onto soluble carriers, extracellular vesicles or lipoprotein particles.

During starvation-induced autophagy in the *Drosophila* fat body, Klp98A was previously found to mediate anterograde autophagosome transport and autophagosome-lysosome fusion in association with Atg8 and Rab14 (Mauvezin et al., 2016). In our study, WgGal4-driven overexpression of Atg8-mCherry and simultaneous knockdown of Klp98A induced early larval lethality, indicating that developmental autophagic processes in Wg-expressing cells also depend on Klp98A. During *Drosophila* eye development, autophagy was recently found to drive cell competition (Nagata et al., 2019). Given that neither larval growth nor wing development was perturbed by Klp98A knockdown alone, apical to basal transport of MVEs during wing imaginal disc development represents a new autophagy-independent function of Klp98A.

Interestingly, Kif16B, the human ortholog to Klp98A, has been found to mediate anterograde motility of early endosomes and recycling of endosomal cargo, such as EGF and transferrin (Hoepfner et al., 2005). Furthermore, in polarized epithelia deficient for the basolateral sorting-adaptor AP-1B, Kif16B was shown to shuttle basolateral proteins, such as the transferrin receptor, towards the apical plasma membrane (Perez Bay et al., 2013). Despite the clear difference of directionality that arises from different orientations of the microtubule network, Kif16B-mediated apical transcytosis in Madin-Darby canine kidney (MDCK) cells and Klp98A-mediated basal transcytosis in the wing imaginal disc epithelium could represent functionally related processes. In MDCK cells, Kif16B mediates transport along non-centrosomal microtubules emerging from the Golgi apparatus towards the apical cell side, whereas non-centrosomal microtubules in the third instar *Drosophila* wing imaginal disc epithelium are clustered at the apical cell side and reach towards basal cellular domains (Mogensen and Tucker, 1987; Tillery et al., 2018). Owing to this inversion, Klp98A-mediated plus end-directed transport results in basal transcytosis as opposed to Kif16B-mediated apical transcytosis. Importantly, alterations in Kif16B function did not only affect endosomal recycling, but also modulated the degradation of the EGF receptor (Hoepfner et al., 2005). This highlights the regulatory role of kinesins, such as Kif16B, at the interface of degradative versus secretory cargo shuttling and points out how kinesin-based transport could mediate the balance between recycling and degradation. It is tempting to speculate that in the complex Wg trafficking network, Klp98A-mediated endosomal transport fine-tunes signal transduction by maintaining a balance between ligand recycling and transcytosis.

## MATERIALS AND METHODS

### Antibodies

The following antibodies and concentrations were used for immunofluorescence staining: Armadillo 1:10 [mouse, N27A1, Developmental Studies Hybridoma Bank (DSHB)], Arrow 1:10,000 (rabbit, a kind gift from Steve DiNardo, University of Pennsylvania, USA), Calnexin 1:10 (mouse, Cnx99A 6-2-1, DSHB), DE-cadherin 1:5 (rat, DCAD2, DSHB), Distalless 1:250 (rabbit, a kind gift from Grace Boekhoff-Falk, University of Wisconsin-Madison, USA), GFP 1:500 (mouse, A11120, and rabbit, A11122, Molecular Probes), GM130 1:250 (rabbit, ab30637, Abcam), Hrs 1:10 (mouse, Hrs8-2 and Hrs27-4, DSHB), Lamp1 1:100 (rabbit, ab30687, Abcam), mCherry 1:1000 (rabbit, ab167453, Abcam), Rab5 1:500 (rabbit, ab31261, Abcam), Rab7 1:10 (mouse, Rab7, DSHB), Sens [rabbit 1:1000, a kind gift from Hugo Bellen (Howard Hughes Medical Institute, Houston, TX, USA)], α-tubulin 1:100 (mouse, 12G10, DSHB), Vestigial 1:100 (rabbit, a kind gift from Kirsten A. Guss, Dickinson College, USA), Wg used 1:3 for extracellular and 1:20 for total staining (mouse, 4D4, DSHB) and Evi 1:500 [rabbit, a kind gift from Konrad Basler (University of Zurich, Switzerland)]. Secondary antibodies directed against the species of interest were coupled to Alexa Fluor 488, 568 and 647 (1:500, Invitrogen).

### *Drosophila* stocks and genetics

The following *Drosophila* stocks were used in this study: *WgGal4* [chromosome II; a kind gift from S. Cohen (University of Copenhagen, Denmark)], WgGal4/CyOtwistGFP; mCherry-Tsp96F/TM6B (K.L., M.H.-C. and J.C.G., unpublished), *UAS-GFP-Myc-2XFYVE* [chromosome III; a kind gift from M. Gonzalez-Gaitan (Wucherpfennig et al., 2003)], Wg-GFP [a kind gift from Filip Port (Port et al., 2014)] and UAS-Lamp1-GFP [a gift from T. Vaccari (Vaccari et al., 2010)]. The following stocks were obtained from Bloomington *Drosophila* Stock Center: *en-Gal4* (#30564), *UAS-Dcr; enGAL4, UAS-GFP* (#25752), *UAS-Rab4-YFP* (#9767) and *UAS-Rab5Q88L-YFP* (#9773). For the kinesin screen in Fig. 1, RNAi lines listed in Table S1 were crossed to WgGal4/CyOtwistGFP; mCherry-Tsp96F/TM6B.

### Immunostaining

Immunostaining of wing imaginal discs was performed according to published protocols. Wing imaginal discs were fixed in 4% PFA in PBS (20 min at room temperature), permeabilized in 0.1% Triton X-100 in PBS and blocked in 5% fetal bovine serum. Primary antibody labeling was performed overnight at 4°C. After washing and secondary antibody labeling, imaginal discs were mounted in Mowiol. Extracellular staining of Wg and Wg-GFP was carried out as previously described (Strigini and Cohen, 2000).

To monitor endocytosis and intracellular trafficking of endocytic cargo, wing imaginal discs were dissected in S2 medium supplemented with 10% fetal bovine serum and incubated with Dextran-A568 (Thermofisher) for the indicated pulse time, rinsed three times in PBS and incubated in supplemented S2 medium for the indicated chase time. To monitor Wg endocytosis and intracellular trafficking, wing imaginal discs were incubated in mouse anti-Wg (1:5, 4D4, DSHB) for the indicated times at 22°C. To remove extracellular antibody signal, wing imaginal discs were rinsed three times in PBS and acid washed in 0.1 M glycine-HCl buffer, pH 3.5 for 30 s at room temperature. Wing imaginal discs were rinsed three more times in PBS before being fixed and stained as described.

For visualization of Wg secretion from living wing imaginal discs, wing imaginal discs were incubated in mouse anti-Wg (1:5, 4D4, DSHB) for 45 min at 22°C before being washed, fixed and stained without any permeabilization. Lysotracker staining was performed using Lysotracker Red DND-99 (Thermofisher) at a dilution of 1:20 for 5 min on ice. Wing imaginal discs were then fixed and stained without permeabilization.

### Microscopy and image analysis

Images were aquired using a Zeiss LSM780 confocal microscope, and *z*-stacks were generated with 0.5 µm intervals using a Plan Neofluar ×63/oil NA 1.4 objective. Confocal images were processed with Zen lite (Zeiss), Fiji/ImageJ (NIH) (Rueden et al., 2017; Schindelin et al., 2012; Schneider et al., 2012) and Affinity Designer (Affinity). Microscopy conditions were kept identical for colocalization analyses and for applications that did not allow posterior to anterior signal normalization. Quantification of colocalization was performed by calculating Pearson's correlation coefficients of background-subtracted *z*-stacks using the Fiji/ImageJ plug-in JaCop (Bolte and Cordelières, 2006). The number of endosomes and size of Rab5Q88L endosomes were quantified manually using Fiji/ImageJ.

### Electron microscopy

Wing imaginal discs were fixed in 2.5% glutaraldehyde in 100 mM phosphate buffer (pH 7.2), washed in 100 mM phosphate buffer and postfixed in 2% osmium tetroxide in phosphate buffer for 1 h on ice. After contrasting en bloc in 2% uranyl acetate, the specimens were dehydrated in EtOH and embedded in araldite using acetone as an intermediate solvent. Thin sections were stained with 2% uranyl acetate and lead citrate. Sections were observed under an EM 109 (Zeiss) microscope at 80 kV. MVE distance to the apical plasma membrane and MVE diameter were quantified manually in Fiji/ImageJ (NIH) (Rueden et al., 2017; Schindelin et al., 2012; Schneider et al., 2012).

### Wing preparation and microscopy

Adult fly wings of respective genotypes were collected in isopropanol, mounted in Hoyer's medium/lactic acid (1:1) and baked overnight at 65°C. Wings were imaged using an Axioplan2 microscope, Fluar 5× NA 0.25 objective and AxioCam HRc camera (Zeiss).

### Statistics

All experiments were carried out at least in biological triplicates. All graphs depict the mean±s.d. Statistical significance was calculated by Student's paired or unpaired two-tailed *t*-test as indicated. Mean values and 95% confidence intervals for all quantified data sets of Figs 1-7 are available in Table S2. In order to confirm true equivalence of Wg target gene expression, a two one-sided test procedure was performed (Burt, 2013; Schuirmann, 1987). An equivalence threshold of 0.25 as an expected relevant difference was chosen based on literature review (e.g. Chaudhary and Boutros, 2019b) and the null hypothesis defined as a difference between control and knockdown above the threshold level.

## Supplementary Material

Supplementary information

Reviewer comments
