# Supplementary material for: The kinesin motor Klp98A mediates apical to basal Wg transport
Source: Development. 2020 Aug 14;147(15):dev186833. doi: 10.1242/dev.186833 (PMC7438014; doi:10.1242/dev.186833)
Supplement: Supplementary information [file develop-147-186833-s1.pdf]

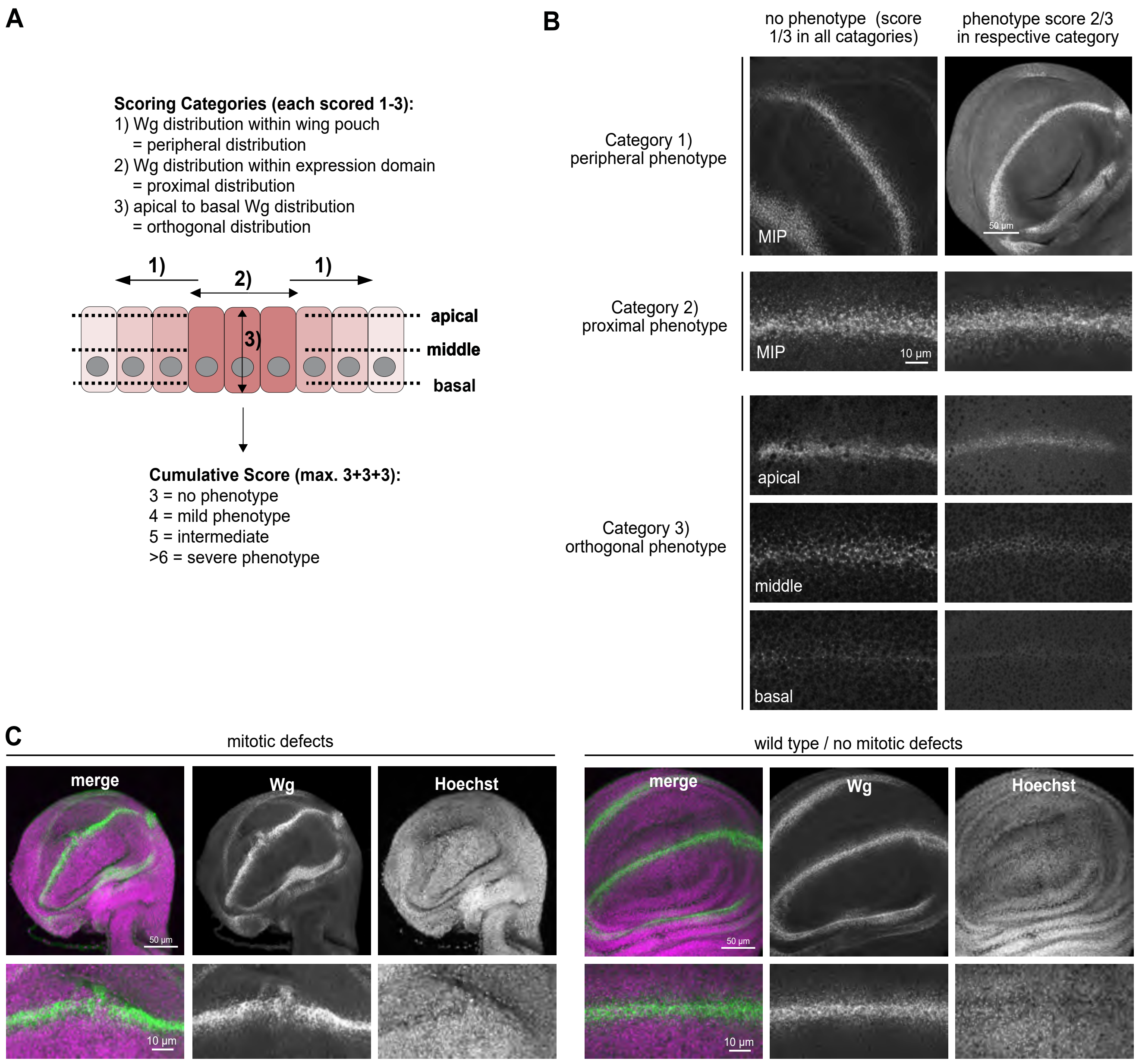

Figure S1: Scoring of Wg phenotypes for in vivo kinesin RNAi Screening.

Figure S1: Scoring of Wg phenotypes for in vivo kinesin RNAi Screening. (A) Schematic representation of the screening approach: Wg phenotypes after WgGal4-driven kinesin knockdown were scored on a scale of one to three in three categories: 1) Wg distribution in the wing pouch (≙ Wg spread/peripheral Wg distribution), 2) Wg distribution within the Wg-expressing cell stripe (≙ secretory Wg trafficking/proximal Wg distribution), 3) Apical to basal Wg distribution within Wg-expressing cells (≙ secretory Wg trafficking/orthogonal Wg distribution). Afterwards, cumulative phenotype scores were calculated and kinesins were grouped according to the resulting cumulative phenotype strength. **(B)** Example images of phenotypes leading to alterations in the peripheral (top), proximal (middle) or orthogonal (bottom) distribution of Wg after WgGal4-driven kinesin knockdown. The depicted phenotypes were scored with a severity of 2 out of 3 in the respective category. The control panel on the left side shows example WID images with no phenotype that were scored with a severity of 1 out of 3. Phenotype scores of 3 out of 3 in the absence of mitotic defects were only observed for Klp98A knockdown. Depicted are MIP of apical to basal z-stacks (top and middle panel) and individual confocal sections from apical, intermediate and basal planes of the WID epithelium (lower panel). **(C)** Example of a mitotic phenotype arising from WgGal4-driven kinesin knockdown marked by alterations in WID morphology.

Development • Supplementary information

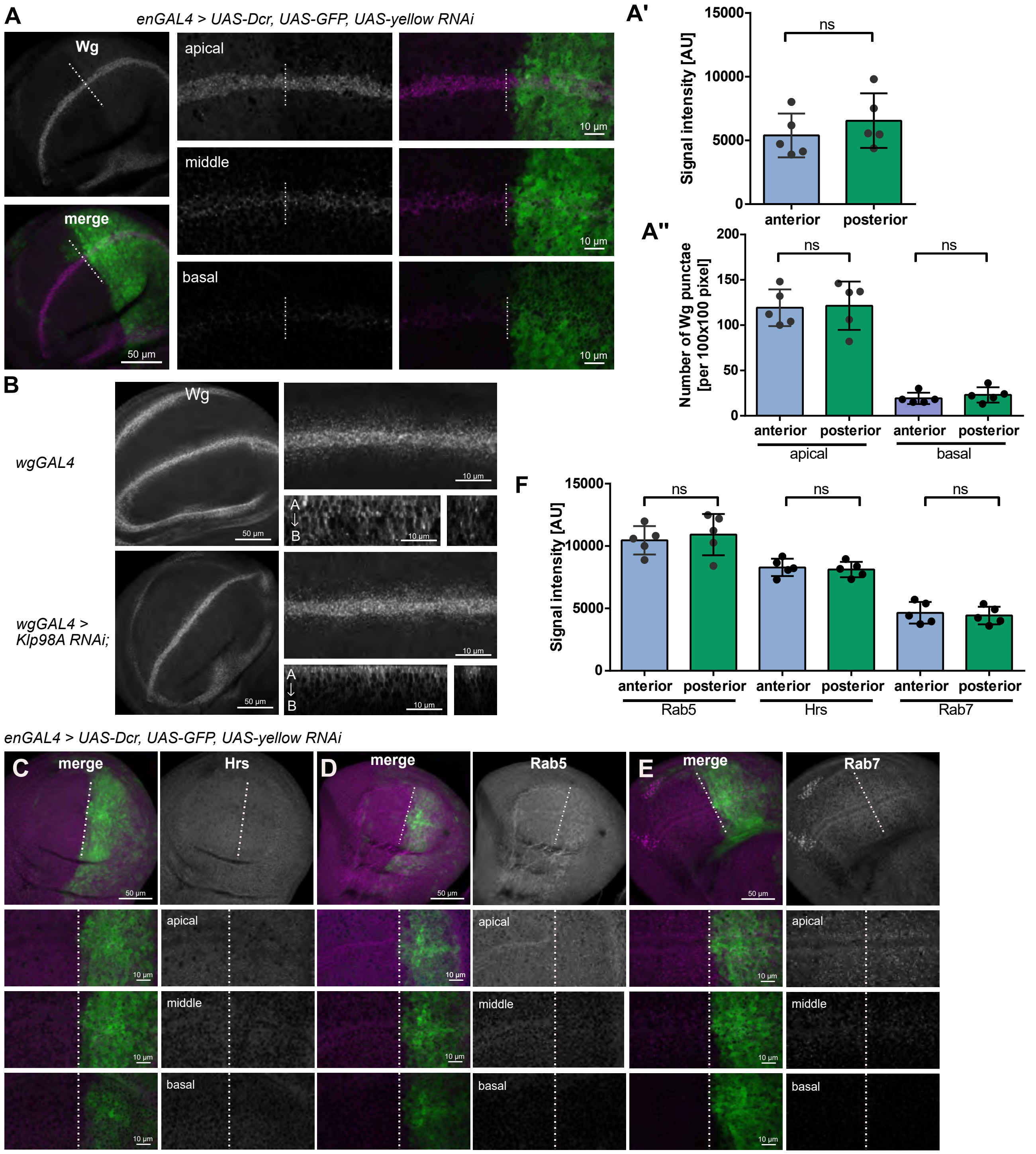

Figure S2: Anterior and posterior wing imaginal discs show no intrinsic differences with regard to Wg or endosomal compartmens.

Figure S2: Anterior and posterior control wing imaginal discs show no intrinsic differences with regard to Wg or endosomal compartments. (A) EnGal4-driven expression of non-targeting yellow RNAi (marked by co-expression of GFP) does not affect Wg distribution in posterior wing imaginal discs. Overview images (left) are MIP of apical to basal *z*-stacks, whereas panels show individual confocal sections from apical, intermediate and basal planes of the wing imaginal disc epithelium. Images are representative of three independent experiments with > six wing imaginal discs. **(A', A")** EnGal4-driven expression of control (yellow) RNAi did not affect overall levels of Wg (A') or apical to basal distribution of punctate Wg (A''). No anterior/posterior differences were observed. Endosome punctae were quantified individually from 100x 100 pixel ROIs in the three most apical sections below the peripodial membrane and three basal sections. **(B)** WgGal4-driven Klp98A knockdown induces apical accumulation of Wg in punctate structures while reducing Wg in basal domains. Overview images and magnifications are MIP of apical to basal *z*-stacks, whereas panels show apical to basal cross-sections of the wing imaginal disc epithelium. Images are representative of three independent experiments with > six wing imaginal discs. **(C-E)** EnGal4-driven expression of non-targeting yellow RNAi (marked by co-expression of GFP) in posterior wing imaginal disc does not interfere with normal localization of Hrs (C), Rab5 (D) or Rab7 (E).The GFP negative compartment serves as an internal control. Overview images are MIP of apical to basal *z*-stacks, whereas panels show individual confocal sections from apical, intermediate and basal planes of the wing imaginal disc epithelium. Images are representative of three independent experiments with > six wing imaginal discs. **(F)** EnGal4-driven expression of control (yellow) RNAi did not affect overall levels of endosomal compartments. Signal intensity was analysed in 100x100pixel ROIs of average intensity projections of anterior and posterior wing imaginal disc separately.

enGAL4 > UAS-Dcr, UAS-GFP, Klp98A RNAi

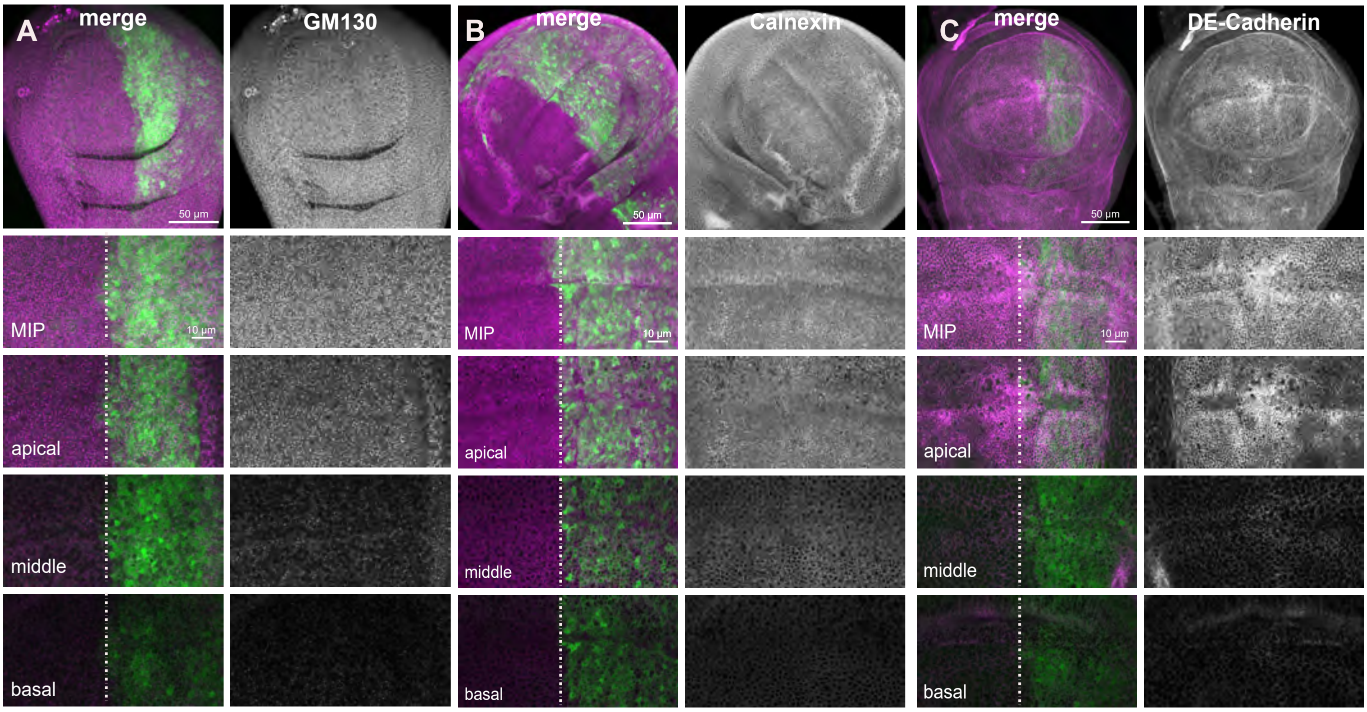

Figure S3: Klp98A knockdown does not affect non-endosomal compartments

Figure S3: Klp98A knockdown Klp98A knockdown does not affect non-endosomal compartments. (A-C) EnGal4-driven Klp98A knockdown in posterior wing imaginal disc (marked by co-expression of GFP) does not interfere with normal localization of Golgi-marker GM130 **(A)**, ER-marker calnexin **(B)** or DE-cadherin **(C)**. Overview images are MIP of apical to basal *z*-stacks, whereas panels show individual confocal sections from apical, intermediate and basal planes of the wing imaginal disc epithelium. Images are representative of three independent experiments with > six wing imaginal discs.

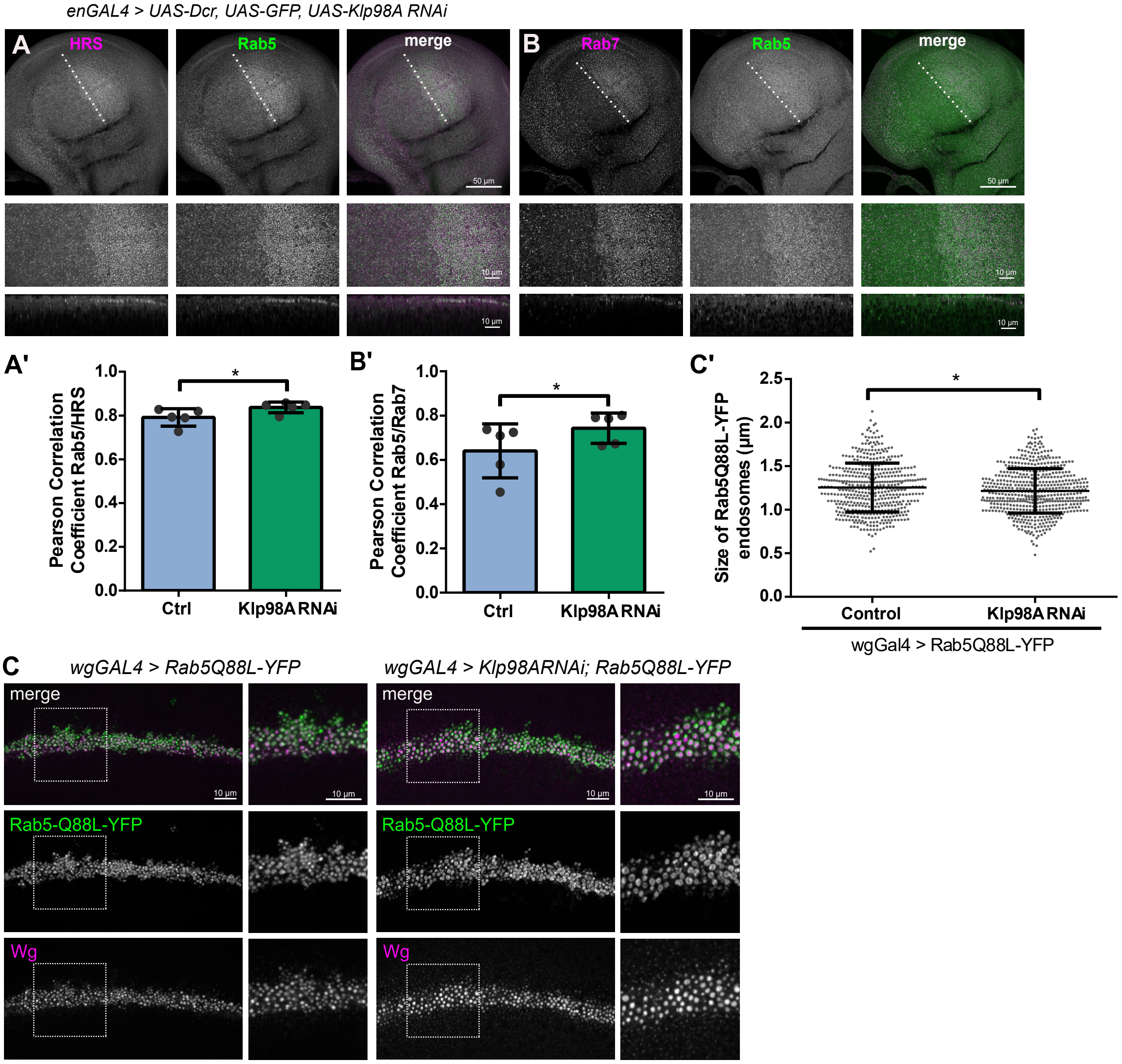

Figure S4: Klp98A knockdown does not interfere with Wg sorting into endosomes

Figure S4: Klp98A knockdown does not interfere with Wg sorting into endosomes. (A, B) Colocalization of Hrs/ Rab5 (A) and Rab7/ Rab5 (B) is increased upon enGal4-driven Klp98A knockdown in posterior wing imaginal disc. Overview images (top) and magnifications (middle) show MIP of apical to basal *z*-stacks, whereas the bottom panel shows optical transverse sections. Images are representative of three independent experiments with > six wing imaginal discs. **(A', B')** Pearson correlation coefficients of Hrs/Rab5 (A') and Rab7/Rab5 (B') are increased upon Klp98 knockdown. Colocalization was analysed in 100x100pixel ROIs in the anterior and posterior wing imaginal disc separately. Paired two-tailed *t*-test, *P*=0.0159 (A), *P*= 0.0205 (B). **(C)** Expression of constitutive active Rab5Q88L-YFP alone or in combination with Klp98A RNAi driven by wgGal4 leads to enlarged endosomal structures that contain Wg. **(C')** Klp98A knockdown reduces the size of Rab5Q88L-enlarged endosomes. The diameter of Rab5Q88l-YFP positive vesicles with a clear lumen was measured in three representative wing imaginal disc from three biological replicates. In total 499, (control) and 573 (Klp98A knockdown) enlarged endosomes were quantified. Two-tailed *t*-test, *P*=0.0188.

Development • Supplementary information

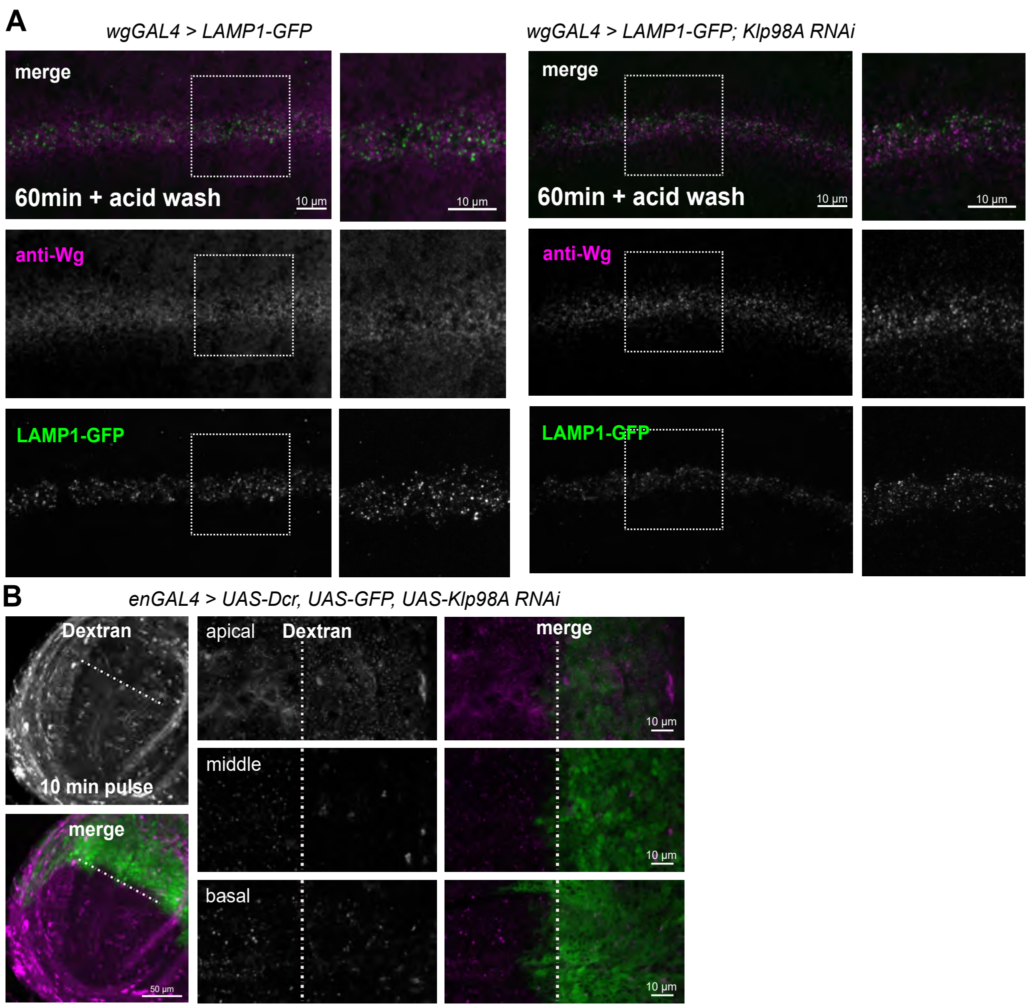

Figure S5: Klp98A knockdown does not interfere with endocytic uptake and does not affect lysosomal Wg transport

Figure S5: Klp98A knockdown does not interfere with endocytic uptake and does not affect lysosomal Wg transport. (A) Endocytosis of anti-Wg antibody in wing imaginal disc expressing GFP-LAMP1 alone (left) or in combination with Klp98A RNAi (right) driven by wgGal4 shows little colocalization of post-endocytic Wg with GFP-LAMP1 in the presence and absence of Klp98A knockdown. Depicted are MIP of apical to basal *z*-stacks. **(B)** Endocytosis of Dextran-A568 after 10min pulse in enGal4-driven Klp98A knockdown wing imaginal disc. Overview images are MIP of apical to basal *z*-stacks, whereas panels show individual confocal sections from apical, intermediate and basal planes of the wing imaginal disc epithelium.

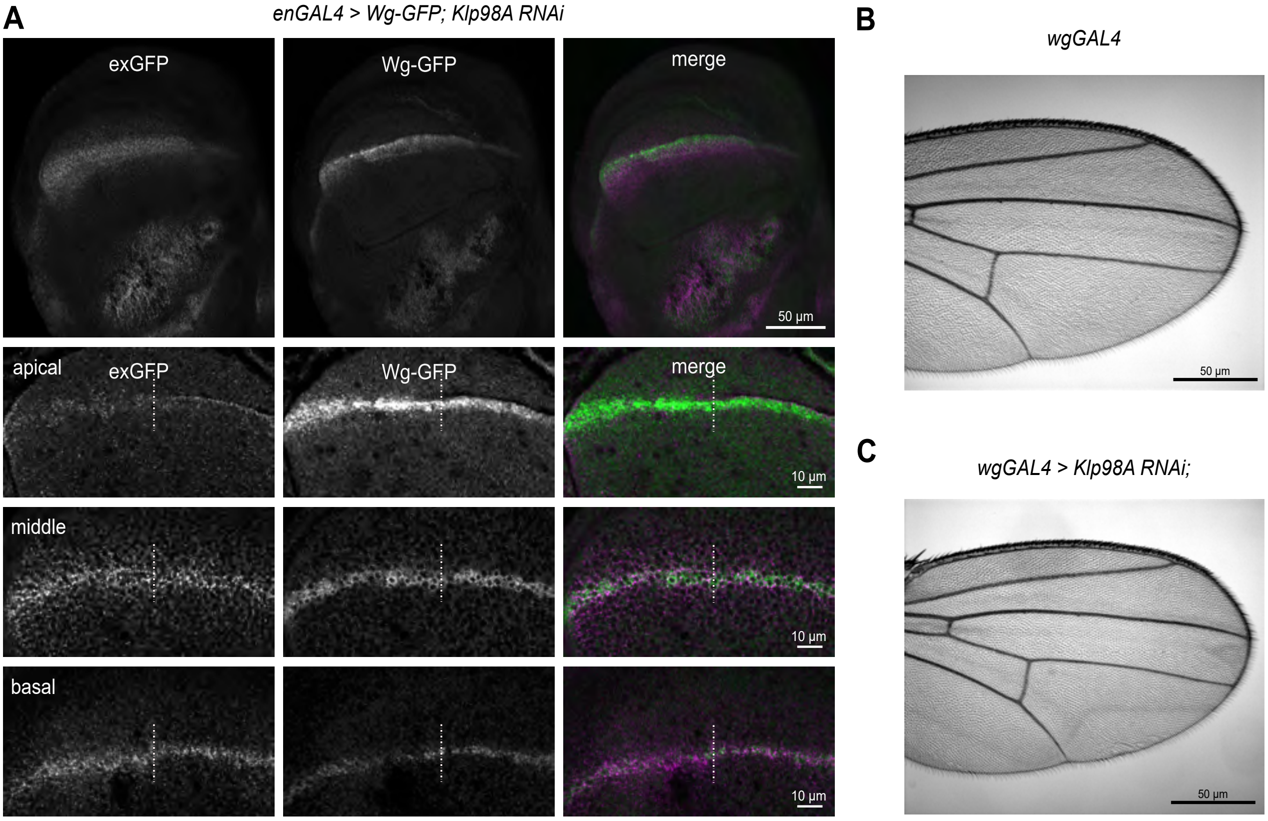

Figure S6: Apical to basal Wg transport mediated by Klp98A is not necessary for normal extracellular Wg levels and wing development

Figure S6: Apical to basal Wg transport mediated by Klp98A is not necessary for normal extracellular Wg levels and wing development. (A) EnGal4-driven Klp98A knockdown in posterior wing imaginal discs does not reduce extracellular levels of endogenous GFP-tagged Wg. Total GFP-Wg (green) is discriminated from extracellular signal by extracellular anti-GFP staining (magenta). MIP (overview) and individual confocal sections from apical, subapical and basal planes of the wing imaginal disc epithelium (magnifications). **(B, C)** WgGal4-driven Klp98A knockdown does not induce wing notches. Representative wings of wild type (B) and Klp98A knockdown (C) flies are shown.

Table S1: Drosophila RNAi lines used for in vivo kinesin RNAi Screening.

Click here to Download Table S1

Table S2: Mean values and 95% confidence intervals for quantified data of Figure 1-7.

Click here to Download Table S2

Development • Supplementary information
