# Supplementary material for: The kinesin motor Klp98A mediates apical to basal Wg transport
Source: Development. 2020 Aug 14;147(15):dev186833. doi: 10.1242/dev.186833 (PMC7438014; doi:10.1242/dev.186833)
Supplement: Reviewer comments [file develop-147-186833_review_history.pdf]

The Kinesin motor Klp98A mediates apical to basal Wg transport

Leonie Witte, Karen Linnemannstöns, Kevin Schmidt, Mona Honemann-Capito, Ferdinand Grawe, Andreas Wodarz and Julia Christina Gross
DOI: 10.1242/dev.186833

Editor: Thomas Lecuit

Review timeline

Original submission:	4 December 2019
Editorial decision:	13 January 2020
First revision received:	11 April 2020
Editorial decision:	12 May 2020
Second revision received:	15 June 2020
Accepted:	2 July 2020

Original submission

First decision letter

MS ID#: DEVELOP/2019/186833

MS TITLE: Kinesin-dependent transcytosis is not necessary for Drosophila Wnt gradient formation

AUTHORS: Leonie Witte, Karen Linnemannstoens, Kevin Schmidt, Mona Honemann-Capito, Ferdinand Grawe, Andreas Wodarz, and Julia Christina Gross

I have now received all the referees' reports on the above manuscript, and have reached a decision. The referees' comments are appended below, or you can access them online: please go to BenchPress and click on the 'Manuscripts with Decisions' queue in the Author Area.

As you will see, the referees express interest in your work, but have some important criticisms and recommend a substantial revision of your manuscript before we can consider publication. If you are able to revise the manuscript along the lines suggested, which may involve further experiments, I will be happy receive a revised version of the manuscript. Your revised paper will be re-reviewed by one or more of the original referees, and acceptance of your manuscript will depend on your addressing satisfactorily the reviewers' major concerns. Please also note that Development will normally permit only one round of major revision.

Please attend to all of the reviewers' comments and ensure that you clearly highlight all changes made in the revised manuscript. Please avoid using 'Tracked changes' in Word files as these are lost in PDF conversion. I should be grateful if you would also provide a point-by-point response detailing how you have dealt with the points raised by the reviewers in the 'Response to Reviewers' box. If you do not agree with any of their criticisms or suggestions please explain clearly why this is so.

Reviewer 1

Advance summary and potential significance to field

Witte et al

© 2020. Published by The Company of Biologists under the terms of the Creative Commons Attribution License (https://creativecommons.org/licenses/by/4.0/).

This manuscript explores the role of Kinesin-mediated transport along microtubules in transcytosis of Wingless (Wg). The authors show that, of all Drosophila kinesins, Klp98A is most strongly required for apical to basal transcytosis of Wg within Rab5+ early endosomes Hrs+ MVBs, and Rab7+ Late endosomes. This is a nice finding, as it provides a mechanism for apical to basal transport of these endosomes. However, the authors do not focus on this nice finding, and instead choose to frame their entire manuscript around the idea that apical to basal transcytosis is not required for Wg signalling, which is a very difficult point to prove. If the authors can revise their manuscript to focus on the positive results "Kinesin-mediated transport of endosomes along microtubules mediates Wg transcytosis", I would recommend publication.

Comments for the author

Major comments

1. Page 2 introduction sets up a big controversy: "While basolateral Wg secretion and spread was proposed as the main source of the active Wg signal (Yamazaki et al., 2016), several publications have demonstrated apical Wg secretion and spreading as responsible for Wg signaling activation (Chang and Sun, 3 2014; Chaudhary and Boutros, 2019; Gallet et al., 2008), thus questioning the contribution of basolateral Wg release to Wg signaling in the WID." But the authors findings do not resolve this controversy, so they should perhaps simply say that Wg is released apically and is also transcytosed basally in the wing disc. Arguments about which secretion route is really essential for signalling are confounded by the fact that long-range release by any route is not strictly necessary due to the early gradient of Wg expression across the entire disc (why NRT-Wg can rescue a wg null mutant). And the authors don't need to solve any of this, so why focus on it?

2. Page 3 introduction: "Here, we analyze the functional relevance of Wg trafficking for apical or basal release by investigating the molecular motors involved in endosomal Wg transport". Again, this goes too far. Just say "Here, we analyse the molecular mechanism of apical-to-basal Wg transport".

3. Page 3 introduction: "Surprisingly, apical to basal Wg transport is not necessary for Wg signal transduction." The authors should remove this sentence because their results do not show this conclusively. The authors could instead say "Apical to basal Wg transport via Klp98A is not strictly necessary for Wg signal transduction, owing to possible redundancy with other kinesins, to parallel mechanisms of Wg secretion, or to the early gradient of Wg expression in this tissue that can bypass the need for secretion and long-range spread of Wg."

4. Results page 9. "Apical-to-basal Wg transcytosis is not necessary for Wg signal propagation". Again too strong. 'Via Klp98A needs to be inserted here'.

5. Figure 6 title states: "Apical-to-basal Wg transcytosis is not necessary for Wg signal propagation". This is again problematic because the authors cannot be sure transcytosis is completely blocked owing to possible redundancy with other kinesins or incomplete knockdown with RNAi or microtubule-independent basal trafficking, etc. The figure should be retitled "Klp98A mediated transport is not strictly essential for Wg signalling."

5. Figure 7 title "Apical Wg recycling is sufficient for signal propagation" is also problematic as the authors cannot be sure that no basal Wg is secreted in these experiments.

Minor comments

1. WID should be spelled out as 'wing imaginal disc' throughout the manuscript.

<u>Reviewer 2</u>

Advance summary and potential significance to field

The molecular mechanisms underlying Wingless/Wnt morphogen secretion and spreading in epithelial tissues is a hot topic of general interest to understand cell proliferation and

© 2020. Published by The Company of Biologists under the terms of the Creative Commons Attribution License (https://creativecommons.org/licenses/by/4.0/).

differentiation. The current theme is that in Drosophila wing imaginal disc, Wg is first transported apically, internalized and targeted to multivesicular endosomes (MVE) for subsequent apical and basal secretion. There is an intense debate in the community to decipher the relative contribution of apical versus basal secretion to Wg long- and short- range signal transduction. Work from the Sun, Boutros's labs argue that Wg secreted and spreading apically is the active Wg signal. Recent work form the Vincent's lab argue that Wg undergoes apical to basal transcytosis and that the pool of Wg secreted basally is the main source of active Wg.
In the present article, Leonie White and colleagues investigate the role of Kinesins in Wg transport and secretion using Drosophila wing imaginal disc as a model system. Using an RNAi based approach they identify the kinesin-3 family member Klp98A (three independent RNAi lines) as a master regulator of Wg intracellular distribution. Silencing of Klp98A is shown to cause the apical mispositionning of Wg-positive endosomes and to control MVE diameter. The authors convincingly demonstrate that Klp98A activity is dispensable for endocytosis and transport of Wg to MVE, dispensable for the gradient of extracellular Wg apically and basally, dispensable for Rab4 dependent fast recycling and dispensable for short and long range Wg signal propagation (Arm, Senseless, Dll and vestigial). Based on the fact that the ortholog of klp98A, KIF16B was reported to promote transcytosis in mammalian epithelial cells, the authors propose a model according which Klp98A regulates the apical to basal transcytosis of Wg. Due to the lack of functional consequences of loss of Klp98A, the authors further propose that transcytosis independent routes for Wg traffic exist and that apical recycling leads to Wg signal propagation upon loss of Klp98A.
The data provided in this manuscript lead to plausible conclusions but in my opinion the conclusions are not fully supported by the experiments. The major criticism resides in the lack of direct assay to monitor transcytosis of Wg (see my specific comments below), namely internalization of Wg from one side of the cell and secretion on the other side of the cell. In the present form, while the data elegantly highlight the function of Klp98A in the positioning of Wg-positive MVE, the data failed to provide sufficient enough mechanistic insights to demonstrate that transcytosis is dispensable and that apical Wg recycling is sufficient for Wg propagation upon loss of Klp98A.

Comments for the author

The imaging, transport assays, and quantitations are state of the art and of very high quality. The experiments and analyses have been carefully conducted. However, I feel that the conclusions are not supported by the experiments for the following reasons. Addressing this point will make the manuscript suitable for publication.
Transcytosis of Wg: the authors did not provide an assay to monitor transcytosis. What is shown is the mispositioning of Wg-positive MVE, the endocytosis of Wg, but nothing about Wg apical basal transportation per se. Of note apical-basal distribution of extracellular Wg is not affected by the loss of Klp98A. If Wg is secreted apically and transcytosis prevented upon loss of Klp98A, the authors should provide some explanation on how extracellular Wg can spread basally in this genetic context. How can the authors formally exclude the possibility that these basal/lateral pools of extracellular Wg could contribute to signal and support their conclusion 'demonstrate that apical Wg recycling is sufficient for Wg transduction in the absence of Klp98A'. Could the author use the tag switching construct (Wg::OLLAS Wg::HA) developed in (Yamachita et al 2016) and do time course experiments to monitor the trafficking of Wg upon loss of Klp98A. Cell surface labeling and chase of Wg to monitor the becoming of internalized Wg and subsequent recycling to the plasma membrane might be an alternative. Alternately, live imaging may help address the point. FRAP of Wg-GFP present in MVE should result in a depletion of apical Wg-GFP spreading without affecting the basal Wg-GFP.

Is the analogy to Kif16B function for transcytosis appropriate? In the article from Rodriguez-Boulan's lab (Perez Bay et al, 2013), the function of Kif16B was revealed in a cell line devoid of the AP-1B complex. Loss of AP-1B prevents basolateral recycling of cargoes, leading to their misrouting to apical recycling endosomes, hence apical delivery. Perez Bay et al have developed very sensitive transcytosis assays based on the use of doubly labelled Tfn. In the genetic context of AP-1B deficient cells, Kif16B was found to be required for apical delivery to ARE, therefore regulate basal to apical transcytosis, the opposite directionality than that proposed in the current manuscript.

© 2020. Published by The Company of Biologists under the terms of the Creative Commons Attribution License (https://creativecommons.org/licenses/by/4.0/).

Reviewer 3

Advance summary and potential significance to field

In the paper entitled "Kinesin-dependent transcytosis is not necessary for Drosophila Wnt gradient formation", Witte et al. show that the kinesin-3 family member Klp98A is responsible for Wingless (Wg) transport following endocytosis. The authors also show that, despite the apical mislocalization of Wg, this has no effect on the extracellular distribution of Wg and its downstream signaling activity.

Comments for the author

As a general comment, the findings of the paper are very interesting, but those sections discussing negative results should be properly presented. Negative results should be given the same importance of positive results: they require proper statistical analyses and convincing experiments. For these reasons we believe this manuscript is not yet suitable for publication in Development.

The authors should address the following points:

Major points

1) The way the authors scored for the severity of the phenotypes in Fig.1C is not clearly explained. They should describe how the phenotype is classified as "severe" vs "mild". The authors should at least provide examples of the different phenotypes in the supplementary figures.
2) The authors need to reconsider the way they quantify their data in Fig. 2 and in all the other figures in which the two compartments are directly compared. A control disc (en-Gal4 UAS-GFP) should be used to compare the difference between the two compartments in the control disc versus the difference between the two compartments (internal control compartment and manipulated posterior compartment). In this way the control would be based on the normalized average of the different data points and not on the individual measurements normalized to 1. This will consequently allow for the calculation of a standard deviation on the control distribution and a proper statistical significance test between the two conditions.
In most experiments the sample size is rather low and the results are statistically relevant because they rely on the fact that one distribution possesses no deviation between its individual measurements.
3) The claims in Fig. 5C-D are not convincing.
4) As this paper is mostly based on negative results, authors should verify if their sample size is appropriate to draw negative conclusions by performing statistical power tests.

Minor points

1) Abbreviations should be properly introduced. For example: Wg should be first introduced as Wingless
(Wg), or MVE should first appear as "Multivescicular endosome (MVE). The authors should choose between using the nomenclature Evi or Wls, we suggest the second. Some abbreviations are not required
(i.e. WID should be "wing imaginal disc" or simply "wing disc").
2) In Figure 1B the scale bar is not present in (3).
3) In Fig. 1E the authors should show a control disc to make sure there is no difference in Wg stripe between the anterior and posterior compartment.
4) Fig. 2B-C are wrongly called in the text.
5) The colocalization assay in Fig.3A' is not convincing. Also, the authors should consider that increased Rab5, Hrs and Rab7 signal in the posterior compartment will increase the colocalization correlation with Wg simply because there is more punctae in that specific compartment.
6) The authors write "neither Wg nor Arrow accumulated" and refer to Fig. 1G, which shows Wg signal intensity in the control vs Klp98A RNAi.
7) The authors should check all their references in the text. (e.g Linnemannstöns et al., DEVELOP/2019/185421)
8) Fig. 4 could become a supplementary figure.

© 2020. Published by The Company of Biologists under the terms of the Creative Commons Attribution License (https://creativecommons.org/licenses/by/4.0/).

9) The authors write “In Klp98E KD WID”, but actually they should refer to the posterior compartment of the wing disc, and not to the wing disc as a whole.
10) It is not clear what Fig.5B should represent and it is not properly explained in the text.
11) Images are not properly aligned in Fig. 5C-D
12) A bold white B is appearing next to the subapical section of Fig. 5C.

First revision

Author response to reviewers' comments

Thank you very much for your encouraging feedback for our manuscript now termed “**Kinesin motor Klp98A mediates apical to basal Wg transport in the WID epithelium**”. Our point- by-point replies are highlighted in blue underneath the particular reviewer comments, changes in the text are highlighted in green. We changed the focus of the manuscript more towards the finding that Klp98A mediates endosomal trafficking from the apical towards the basal side and performed several additional experiments confirming our previous argumentation.
These include:

- A recycling assay of Wg
- yellow knockdown controls are confirming the previous findings/quantification

We hope to have achieved significant improvement of our manuscript to make it now suitable for publication in Development.

Reviewer 1 Advance Summary and Potential Significance to Field:
Witte et al
This manuscript explores the role of Kinesin-mediated transport along microtubules in transcytosis of Wingless (Wg). The authors show that, of all Drosophila kinesins, Klp98A is most strongly required for apical to basal transcytosis of Wg within Rab5+ early endosomes, Hrs+ MVBs, and Rab7+ Late endosomes. This is a nice finding, as it provides a mechanism for apical to basal transport of these endosomes. However, the authors do not focus on this nice finding, and instead choose to frame their entire manuscript around the idea that apical to basal transcytosis is not required for Wg signalling, which is a very difficult point to prove. If the authors can revise their manuscript to focus on the positive results “Kinesin-mediated transport of endosomes along microtubules mediates Wg transcytosis”, I would recommend publication.

Reviewer 1 Comments for the Author:
Major comments
1. Page 2 introduction sets up a big controversy: “While basolateral Wg secretion and spread was proposed as the main source of the active Wg signal (Yamazaki et al., 2016), several publications have demonstrated apical Wg secretion and spreading as responsible for Wg signaling activation (Chang and Sun, 3 2014; Chaudhary and Boutros, 2019; Gallet et al., 2008), thus questioning the contribution of basolateral Wg release to Wg signaling in the WID.” But the authors findings do not resolve this controversy, so they should perhaps simply say that Wg is released apically and is also transcytosed basally in the wing disc. Arguments about which secretion route is really essential for signalling are confounded by the fact that long-range release by any route is not strictly necessary due to the early gradient of Wg expression across the entire disc (why NRT-Wg can rescue a wg null mutant). And the authors don’t need to solve any of this, so why focus on it?

We thank the reviewer for his/her constructive feedback and change the title and text accordingly to represent better our findings that Klp98A transports Wg-endosomes from the apical towards the basal plasma membrane. In detail, we changed the title to “Kinesin motor Klp98A mediates apical to basal Wg transport in the WID epithelium” and rephrased lines 55-77, 92- 95 and 326-337.

© 2020. Published by The Company of Biologists under the terms of the Creative Commons Attribution License (https://creativecommons.org/licenses/by/4.0/).

2. *Page 3 introduction: "Here, we analyze the functional relevance of Wg trafficking for apical or basal release by investigating the molecular motors involved in endosomal Wg transport". Again, this goes too far. Just say "Here, we analyse the molecular mechanism of apical-to-basal Wg transport".*

We changed the text accordingly. The changes are highlighted in the text, see line 76-77.

3. *Page 3 introduction: "Surprisingly, apical to basal Wg transport is not necessary for Wg signal transduction." The authors should remove this sentence because their results do not show this conclusively. The authors could instead say "Apical to basal Wg transport via Klp98A is not strictly necessary for Wg signal transduction, owing to possible redundancy with other kinesins, to parallel mechanisms of Wg secretion, or to the early gradient of Wg expression in this tissue that can bypass the need for secretion and long-range spread of Wg."*

We changed the text accordingly, see line 92-95.

4. *Results page 9. "Apical-to-basal Wg transcytosis is not necessary for Wg signal propagation". Again too strong. 'Via Klp98A needs to be inserted here'.*

We changed the text accordingly, see line 270.

5. *Figure 6 title states: "Apical-to-basal Wg transcytosis is not necessary for Wg signal propagation". This is again problematic because the authors cannot be sure transcytosis is completely blocked owing to possible redundancy with other kinesins or incomplete knockdown with RNAi or microtubule-independent basal trafficking, etc. The figure should be retitled "Klp98A mediated transport is not strictly essential for Wg signalling."*

We changed the figure title to "Klp98A-mediated apical-to-basal Wg transport is not strictly essential for Wg signal propagation" (line 270).

5. *Figure 7 title "Apical Wg recycling is sufficient for signal propagation" is also problematic as the authors cannot be sure that no basal Wg is secreted in these experiments.*

We changed the figure title to "Klp98A-independent Wg recycling is sufficient for signal propagation".

Minor comments

1. *WID should be spelled out as 'wing imaginal disc' throughout the manuscript.*

We changed the text accordingly.

Reviewer 2 Advance Summary and Potential Significance to Field:
The molecular mechanisms underlying Wingless/Wnt morphogen secretion and spreading in epithelial tissues is a hot topic of general interest to understand cell proliferation and differentiation. The current theme is that in Drosophila wing imaginal disc, Wg is first transported apically, internalized and targeted to multivesicular endosomes (MVE) for subsequent apical and basal secretion. There is an intense debate in the community to decipher the relative contribution of apical versus basal secretion to Wg long- and short- range signal transduction. Work from the Sun, Boutros's labs argue that Wg secreted and spreading apically is the active Wg signal. Recent work form the Vincent's lab argue that Wg undergoes apical to basal transcytosis and that the pool of Wg secreted basally is the main source of active Wg.
In the present article, Leonie White and colleagues investigate the role of Kinesins in Wg transport and secretion using Drosophila wing imaginal disc as a model system. Using an RNAi based approach they identify the kinesin-3 family member Klp98A (three independent RNAi lines) as a master regulator of Wg intracellular distribution. Silencing of Klp98A is shown to cause the apical mispositionning of Wg-positive endosomes and to control MVE diameter. The authors convincingly demonstrate that Klp98A activity is dispensable for endocytosis and transport of Wg to MVE, dispensable for the gradient of extracellular Wg apically and basally, dispensable for

© 2020. Published by The Company of Biologists under the terms of the Creative Commons Attribution License (https://creativecommons.org/licenses/by/4.0/).

Rab4 dependent fast recycling and dispensable for short and long range Wg signal propagation (Arm, Senseless, Dll and vestigial). Based on the fact that the ortholog of klp98A, KIF16B was reported to promote transcytosis in mammalian epithelial cells, the authors propose a model according which Klp98A regulates the apical to basal transcytosis of Wg. Due to the lack of functional consequences of loss of Klp98A, the authors further propose that transcytosis independent routes for Wg traffic exist and that apical recycling leads to Wg signal propagation upon loss of Klp98A.The data provided in this manuscript lead to plausible conclusions but in my opinion the conclusions are not fully supported by the experiments. The major criticism resides in the lack of direct assay to monitor transcytosis of Wg (see my specific comments below), namely internalization of Wg from one side of the cell and secretion on the other side of the cell. In the present form, while the data elegantly highlight the function of Klp98A in the positioning of Wg-positive MVE, the data failed to provide sufficient enough mechanistic insights to demonstrate that transcytosis is dispensable and that apical Wg recycling is sufficient for Wg propagation upon loss of Klp98A.

Reviewer 2 Comments for the Author:
The imaging, transport assays, and quantitations are state of the art and of very high quality. The experiments and analyses have been carefully conducted. However, I feel that the conclusions are not supported by the experiments for the following reasons. Addressing this point will make the manuscript suitable for publication.
Transcytosis of Wg: the authors did not provide an assay to monitor transcytosis. What is shown is the mispositioning of Wg- positive MVE, the endocytosis of Wg, but nothing about Wg apical basal transportation per se. Of note apical-basal distribution of extracellular Wg is not affected by the loss of Klp98A. If Wg is secreted apically and transcytosis prevented upon loss of Klp98A, the authors should provide some explanation on how extracellular Wg can spread basally in this genetic context. How can the authors formally exclude the possibility that these basal/lateral pools of extracellular Wg could contribute to signal and support their conclusion 'demonstrate that apical Wg recycling is sufficient for Wg transduction in the absence of Klp98A'.
Could the author use the tag switching construct (Wg::OLLAS Wg::HA) developed in (Yamachita et al 2016) and do time course experiments to monitor the trafficking of Wg upon loss of Klp98A. Cell surface labeling and chase of Wg to monitor the becoming of internalized Wg and subsequent recycling to the plasma membrane might be an alternative.
Alternately, live imaging may help address the point. FRAP of Wg-GFP present in MVE should result in a depletion of apical Wg-GFP spreading without affecting the basal Wg-GFP.

We thank the reviewer for his/her constructive feedback and suggestions. We agree that, while we conclusively demonstrate that apical to basal transport of endosomal Wg depends on Klp98A, direct visualization of secondary Wg secretion from said compartments would greatly enhance our conclusions. In order to visualize Klp98A-independent apical Wg secretion in our model system, we have now included a novel live staining assay in novel Figure 7, for which wing imaginal discs were incubated 45 min with anti-Wg antibody at 22°C. At this temperature Wg uptake and recycling continues *ex vivo* and extracellular Wg can be antibody-labelled on the surface. In this assay we were able to show that Klp98A knockdown increases the apical levels of extracellular Wg but does not affect basal levels. This assay provides an additional readout for extracellular Wg levels and allows conclusions regarding the dynamic secretion of Wg as opposed to the "steady-state" snapshot of conventional extracellular staining at 4°C.
To further visualize secondary Wg secretion at the apical or basal cell side, we have aimed to establish several additional assay. Firstly, we tried to establish an assay to visualize secondary Wg secretion after antibody-labelling of a surface-bound pool of Wg (pulse) and a consecutive chase. In order to eliminate signal stemming from Wg after primary secretion, Wg epitopes were blocked with unconjugated nanobodies targeting the primary anti-Wg antibody. Due to insufficient blocking capabilities of the nanobody and overall low signal intensities, this assay did however not yield the respective results. In a second attempt, we pulsed with a fluorescently labelled anti-Wg AB and tried to remove surface-bound tagged-Wg by acid- wash in order to enable secondary-antibody labelling of resurfaced Wg after the chase. However, this approach also did not allow us to label reliably secondarily secreted Wg as opposed to stagnant surface-bound or primarily secreted Wg. In order to establish reliable assays for the monitoring of transcytosis and recycling in our model system, more time and experiments would be needed.
Likewise, while we acquired the suggested tag switching construct (Wg::OLLAS Wg::HA) (Yamazaki et al,. 2016), the required generation of UAS-Flp-expressing fly stems and the conduction of time

© 2020. Published by The Company of Biologists under the terms of the Creative Commons Attribution License (https://creativecommons.org/licenses/by/4.0/).

course experiments was beyond the time-scope of this revision (and further aggravated by the current working conditions).
As we were therefore unable to provide sufficient enough mechanistic insights to demonstrate that basal Wg secretion is fully dispensable and that apical Wg recycling is sufficient for Wg propagation, we changed the focus of our manuscript towards the finding that Klp98A mediates endosomal Wg trafficking from the apical towards the basal side and softened our conclusions regarding the role of Rab4-mediated Wg recycling (lines 55-77, 92-95 and 326-337).
We furthermore agree with the reviewer that non-endosomal Wg trafficking, e.g. via direct post-Golgi routes independently of Klp98A could contribute to basal extracellular Wg levels, even though according to our data, only very low levels of Wg were detected intracellularly in basal domains.

Is the analogy to Kif16B function for transcytosis appropriate? In the article from Rodriguez-Boulan's lab (Perez Bay et al, 2013), the function of Kif16B was revealed in a cell line devoid of the AP-1B complex. Loss of AP-1B prevents basolateral recycling of cargoes, leading to their misrouting to apical recycling endosomes, hence apical delivery. Perez Bay et al have developed very sensitive transcytosis assays based on the use of doubly labelled Tfn. In the genetic context of AP-1B deficient cells, Kif16B was found to be required for apical delivery to ARE, therefore regulate basal to apical transcytosis, the opposite directionality than that proposed in the current manuscript.

We agree with the reviewer that the direction of Klp98A-mediated endosomal transport is surprising, as one would expect to see perinuclear cargo accumulation after knockdown of a plus-end directed motor-protein. Clearly, the direction of kinesin- mediated transport depends on the directionality and organization of the cellular microtubule network and cargo accumulation would be expected to occur at microtubule minus ends near the microtubule organizing center (MTOC). In the *Drosophila* wing imaginal disc epithelium of wandering third instar larvae, microtubules are anchored at the apical cell side in non-centrosomal MTOCs and the microtubule network follows a distinct apical to basal polarity (Tillery et al., 2018, Mogensen et al. 1989).
We performed alpha-tubulin staining of wing imaginal discs and, in line with the mentioned publications, detected dense tubulin accumulations at the apical WID cell side that likely correspond to MTOCs (see below). Apical anchorage, and structure of microtubules was not affected by Klp98A knockdown, indicating that Klp98A knockdown does not interfere with microtubule organization. (Interestingly, during pupal wing development, microtubule organization shifts from this apical-basal polarity towards a proximal-distal polarity that is required for polarized transport of PCP proteins (Matis et al., 2014). Due to proper wing development in the absence of Klp98A, we do not expect Klp98A to fulfill critical functions during pupal proximal- distal transport processes.) Due to this specificity of developmental MT organization, the MT network of the Drosophila WID epithelium is inverted as compared to the MDCK cell system used by Rodriguez-Boulan's lab, where non-centrosomal MTs emerge from the (more basally located) golgi apparatus towards the apical cell side. Under these circumstances, we believe that the apical transcytosis mediated by Kif16b and the basal transcytosis mediated by Klp98A can be compared on a functional level. We thank the reviewer for pointing out the need for more explanation for this comparison and have added a corresponding paragraph in the discussion section, see line 430-445.

Marisa M. L. Tillery. Centrosomal and Non-Centrosomal Microtubule-Organizing Centers (MTOCs) in Drosophila melanogaster. Cells. 2018 Aug 28;7(9). pii: E121.
Mogensen, M.M.; Tucker, J.B. Evidence for microtubule nucleation at plasma membrane-associated sites in Drosophila. J. Cell Sci. 1987, 88, 95–107.
Mogensen, M.M.; Tucker, J.B.; Stebbings, H. Microtubule polarities indicate that nucleation and capture of microtubules occurs at cell surfaces in Drosophila. J. Cell Biol. 1989, 108, 1445–1452.
Matis, M.; Russler-Germain, D.A.; Hu, Q.; Tomlin, C.J.; Axelrod, J.D. Microtubules provide directional information for core PCP function. Elife 2014, 3, e02893.

We have removed unpublished data provided for the referees in confidence.

Reviewer 3 Advance Summary and Potential Significance to Field:
In the paper entitled "Kinesin-dependent transcytosis is not necessary for Drosophila Wnt gradient formation", Witte et al. show that the kinesin-3 family member Klp98A is responsible

© 2020. Published by The Company of Biologists under the terms of the Creative Commons Attribution License (https://creativecommons.org/licenses/by/4.0/).

for Wingless (Wg) transport following endocytosis. The authors also show that, despite the apical mislocalization of Wg, this has no effect on the extracellular distribution of Wg and its downstream signaling activity.

Reviewer 3 Comments for the Author:
As a general comment, the findings of the paper are very interesting, but those sections discussing negative results should be properly presented. Negative results should be given the same importance of positive results: they require proper statistical analyses and convincing experiments. For these reasons we believe this manuscript is not yet suitable for publication in Development.
The authors should address the following points:
Major points
1) The way the authors scored for the severity of the phenotypes in Fig.1C is not clearly explained. They should describe how the phenotype is classified as "severe" vs "mild". The authors should at least provide examples of the different phenotypes in the supplementary figures.

We agree with the reviewer and now explain the additive scoring of the screen in more detail in the text (line 108-121) and in novel Supplementary Figure 1A-C and respective figure legend.

2) The authors need to reconsider the way they quantify their data in Fig. 2 and in all the other figures in which the two compartments are directly compared. A control disc (en-Gal4 UAS-GFP) should be used to compare the difference between the two compartments in the control disc versus the difference between the two compartments (internal control compartment and manipulated posterior compartment). In this way the control would be based on the normalized average of the different data points and not on the individual measurements normalized to 1. This will consequently allow for the calculation of a standard deviation on the control distribution and a proper statistical significance test between the two conditions. In most experiments the sample size is rather low and the results are statistically relevant because they rely on the fact that one distribution possesses no deviation between its individual measurements.

We thank the reviewer for this constructive feedback. We have included an enGal4, UAS-Dcr, UAS-GFP x yellow RNAi control in neither novel Supplementary Figure 2, that shows that levels nor positioning of Wg and endosomal compartments show anterior vs. posterior differences in control discs. As suggested, we have compared anterior/posterior ratios for Wg and endosomal signal intensity between control and enGal4-driven knockdown wing imaginal discs and were able to confirm our previous findings (see below). Additional controls were generated using enGal4-driven expression of yellow control RNAi. For data normalization, signals were quantified in the anterior and posterior compartments of x yellow control and x Klp98A RNAi WID independently and anterior/posterior signal ratios were calculated for each WID independently. Expectedly, data obtained by this way of quantification mimicked the results generated by normalization to the control (fold-change). In additional Figure 1, we depict the expected increase in apical and decrease in basal Wg punctae (corresponding to Fig. 1F) as well as Rab5, Hrs and Rab7 (corresponding to Fig. 2A'-C') Importantly, EnGal4-driven expression of yellow did not affect Wg or endosome positioning or signal intensity (see novel supplementary Figure S2) and confirmed that there are no intrinsic anterior/posterior differences in the distribution of Wg or endosomal compartments.

We have removed unpublished data provided for the referees in confidence.

To eliminate the need for data normalization, we have furthermore quantified organelle punctae whenever possible (see Fig. 1F,2A'-C',3A-C'). To follow the reviewers suggestion, we have changed the respective figures to represent non-normalized punctae values instead of normalized data (see Fig. 1F,2A'-C',3A-C') . Whenever necessary due to intrinsic anterior/posterior differences, we have analyzed control and knockdown wing imaginal discs separately as suggested and represented data as a comparison of anterior/posterior ratios between control and knockdown discs (see senseless, distalless and vestigial in Figure 6). As the expected variation in staining intensity and imaging in whole wing imaginal discs is high, the advantage of the well- established enGal4-driven knockdown system lies in the fact that the anterior side of enGAL4 WID can serve as an internal control. Anterior/posterior fold-change furthermore represents an established way of

© 2020. Published by The Company of Biologists under the terms of the Creative Commons Attribution License (https://creativecommons.org/licenses/by/4.0/).

data representation and is seen in a recent Wg paper (Chaudhary and Boutros Development 2019). In summary, albeit the statistical disadvantage of losing variance in the control, we consider the fold change of the RNAi side over several discs to be a good indication for a robust biological effect. For quantification of Wg signal intensities [AU], that are not affected by anterior/posterior differences as described above, we have normalized our data to the respective anterior control side (set to 1).

3) The claims in Fig. 5C-D are not convincing.

We apologize for the insufficient image quality and have updated Fig. 5 to depict clearer images of the Dextran uptake assay (novel Fig.5B). We additionally depict Wg and Dextran trafficking in novel Fig. 5C now. Furthermore, we have moved previous Fig. 5C to Fig. S4B.

4) As this paper is mostly based on negative results, authors should verify if their sample size is appropriate to draw negative conclusions by performing statistical power tests.

We agree with the reviewer, that it is important to consider the number of experiments that lead to negative results. As the wing imaginal disc is a well characterized model system for morphogen trafficking research, we performed experiments in several crosses and dissected 6-10 different imaginal discs for our conclusions, in addition to having an internal control within each tissue. With these replicates we are able to observe significant differences in Klp98A knockdown in respect to Wg localization and endosomal trafficking. However, other markers are not significantly different. While the computation of statistical power after obtaining statistically non-significant results is possible, such computations using the observed small differences between two groups would by definition report low power values (see e.g. Hoenig and Heisley, 2001). Instead, we have included mean values and 95% confidence intervals in Supplementary Table 2 for a clearer data representation. We hope that this is sufficient to enable readers to put our data into context and allows a better interpretation of our results. (Hoenig JM, Heisey DM, 1710, The abuse of power, The American Statistician. February 1, 2001, 55(1): 19- 24.doi:10.1198/000313001300339897.)

Minor points

1) Abbreviations should be properly introduced. For example: Wg should be first introduced as Wingless (Wg), or MVE should first appear as "Multivescicular endosome (MVE). The authors should choose between using the nomenclature Evi or Wls, we suggest the second. Some abbreviations are not required (i.e. WID should be "wing imaginal disc" or simply "wing disc").

We changed the text accordingly.

2) In Figure 1B the scale bar is not present in (3).

We corrected this error.

3) In Fig. 1E the authors should show a control disc to make sure there is no difference in Wg stripe between the anterior and posterior compartment.

We added a Yellow RNAi control WID and show now in Supplementary Figures S1D and S2D-E, that there is no significant difference between anterior and posterior compartments in enGal-driven control RNAi WID with regard to Wg and endosomal compartments.

4) Fig. 2B-C are wrongly called in the text.

We apologize for this error and corrected the text accordingly.

5) The colocalization assay in Fig.3A' is not convincing. Also, the authors should consider that increased Rab5, Hrs and Rab7 signal in the posterior compartment will increase the colocalization correlation with Wg simply because there is more punctae in that specific compartment.

We have requantified the colocalization of Fig. 3A and now depict the percentage of Wg punctae colocalizing with Rab5 instead of Pearson's Coefficient. We furthermore agree with the author's

© 2020. Published by The Company of Biologists under the terms of the Creative Commons Attribution License (https://creativecommons.org/licenses/by/4.0/).

opinion that the increased colocalization could stem from increased signal density and added a respective paragraph to the text (lines 166-168).

6) The authors write "neither Wg nor Arrow accumulated" and refer to Fig. 1G, which shows Wg signal intensity in the control vs Klp98A RNAi.

We apologized for this confusion and call the respective figure more clearly now.

7) The authors should check all their references in the text. (e.g Linnemannstöns et al., DEVELOP/2019/185421)

This refers to a paper submitted to Development as a connected submission (DEVELOP/2019/185421).

8) Fig. 4 could become a supplementary figure.

As endosomal transport is generally directed into one of two branches, namely secretion or degradation, we feel that keeping Fig. 4 as a main figure enhances readability and clarity of this manuscript.

9) The authors write "In Klp98E KD WID", but actually they should refer to the posterior compartment of the wing disc, and not to the wing disc as a whole.

We apologized for this omission and changed the text accordingly (lines 235).

10) It is not clear what Fig.5B should represent and it is not properly explained in the text.

We apologize for this lack of clarity. We have rephrased the respective section and hope that it is now understandable (see lines 238- 245).

11) Images are not properly aligned in Fig. 5C-D

The image depicted a maximum intensity projection and the AP border is not perfectly straight in the xz plane, this is why the line is shifting in the three sections. We made sure that the images themselves are properly aligned.

12) A bold white B is appearing next to the subapical section of Fig. 5C.

We corrected this error.

<u>Second decision letter</u>

MS ID#: DEVELOP/2019/186833

MS TITLE: Kinesin motor Klp98A mediates apical to basal Wg transport in the WID epithelium

AUTHORS: Leonie Witte, Karen Linnemannstoens, Kevin Schmidt, Mona Honemann-Capito, Ferdinand Grawe, Andreas Wodarz, and Julia Christina Gross

I have now received all the referees reports on the above manuscript, and have reached a decision. The referees' comments are appended below, or you can access them online: please go to BenchPress and click on the 'Manuscripts with Decisions' queue in the Author Area.

The overall evaluation is positive and we would like to publish a revised manuscript in Development, provided that the referees' comments can be satisfactorily addressed. I concur with reviewer 3 under their comment 4 regarding the need to perform statistical power test to

© 2020. Published by The Company of Biologists under the terms of the Creative Commons Attribution License (https://creativecommons.org/licenses/by/4.0/).

determine the sample size needed for the statistical tests. I propose to increase the sample size for a given Ab staining to the point where it matches the expected sample size from power test. This will ensure the solidity of the conclusions based on negative results. Please attend to all of the reviewers' comments in your revised manuscript and detail them in your point-by-point response.

We are aware that you may currently be unable to access the lab to undertake experimental revisions. If it would be helpful, we encourage you to contact us to discuss your revision in greater detail. Please send us a point-by-point response indicating where you are able to address concerns raised (either experimentally or by changes to the text) and where you will not be able to do so within the normal timeframe of a revision. We will then provide further guidance. Please also note that we are happy to extend revision timeframes as necessary.

Reviewer 1

Advance summary and potential significance to field

The paper reveals new insights into the mechanism of apical-to-basal Wg trafficking.

Comments for the author

I am satisfied with the revised manuscript, and would suggest only one thing: to remove the words 'in the WID epithelium' from the title. In general, shorter titles are much more effective in attracting readers and citations. Also, I'm not sure anyone will know what WID stands for when they see the title.

Reviewer 2

Advance summary and potential significance to field

This paper identifies and describes the function of Klp98, a kinesin-3 family member, in the polarized endosomal trafficking of Wg in Drosophila epithelium.

Comments for the author

In this revised version, the authors have taken into consideration my comments and modified the text and the focus of their Manuscript. I congratulate them for this work and I believe that in its present form the article can be considered for publication at Development. I only have minor suggestions for improvement.

1- To specifically address my first concern, the authors have developed and included an assay to live-visualize apical and basal Wg secretion in wing imaginal discs. These new data are presented in Figure 7. For Figure 7C, I recommend the authors to specify the number of independent experiments they have conducted and to draw on the image the region of interest considered for quantitation, as it is hard to appreciate what a ROI of 150x150 represents (and also because on panel C, part of the basal section is black, apparently out of focus). Together with the data on Rab4-positive compartment, it provides evidence that Wg can be recycled apically. These data support the conclusion that ‘Klp98A-independent Wg recycling is sufficient for signal propagation’. They have amended the title and the text of their manuscript accordingly.

2- In the discussion section, they have now explained in detail the analogy between Klp98A and Kif16B functions. They hey also provided a reviewer figure illustrating the fact that microtubule organization is not impacted by Klp98A silencing. Nonetheless, at this magnification, the distribution of MT differs in anterior versus posterior compartment. It would have been a good idea to show control discs for comparison. Also, it would have been interesting to show MT +ends (using EB1) at the stage analyzed for Wg secretion.

© 2020. Published by The Company of Biologists under the terms of the Creative Commons Attribution License (https://creativecommons.org/licenses/by/4.0/).

Reviewer 3

Advance summary and potential significance to field

See previous round of reviews.

Comments for the author

The authors have successfully addressed the majority of my points. However, there is one remaining major point (4) that hasn't been addressed by the authors and should be addressed instead. If the author address this point, I believe the paper can be published in Development.
In my point 4), I asked the authors to increase the sample size to demonstrate the validity of their negative conclusions. The authors however responded with 3 arguments:

1) "As the wing imaginal disc is a well characterized model system for morphogen trafficking research, we performed experiments in several crosses and dissected 6-10 different imaginal discs for our conclusions".

I disagree with this statement. Proper statistical tests are specifically designed to define what is the minimum number of discs needed to reach a given conclusion. In addition, dissecting more than 6 to 10 discs is a task requiring about two weeks of work.

2) "With these replicates we are able to observe significant differences in Klp98A knockdown in respect to Wg localization and endosomal trafficking. However, other markers are not significantly different."

This is not a logically valid point.

3) "we have included mean values and 95% confidence intervals in Supplementary Table 2 for a clearer data representation"

This is another way of presenting the same data, which has no relation to whether the sample size is appropriate or not. In addition, the table is not precise (fore example one average is out of the 95% confidence interval), and in some cases, as expected, the 95% confidence intervals are very large.

Second revision

Author response to reviewers' comments

Thank you very much for the handling of our manuscript DEVELOP/2019/186833 and for your encouraging feedback.

We have now changed our manuscript title to “Kinesin motor Klp98A mediates apical to basal Wg transport” as suggested by reviewer 1 and have adapted figure 7 and corresponding figure legends as suggested by reviewer 2. Our point-by-point replies are highlighted in blue underneath the particular reviewer comments, changes in the text are highlighted in green.

Reviewer 1 Advance Summary and Potential Significance to Field:
The paper reveals new insights into the mechanism of apical-to-basal Wg trafficking.

Reviewer 1 Comments for the Author:
I am satisfied with the revised manuscript, and would suggest only one thing: to remove the words 'in the WID epithelium' from the title. In general, shorter titles are much more effective in attracting readers and citations. Also, I'm not sure anyone will know what WID stands for

© 2020. Published by The Company of Biologists under the terms of the Creative Commons Attribution License (https://creativecommons.org/licenses/by/4.0/).

when they see the title.

We thank the reviewer for this evaluation and have changed to title as suggested to "Kinesin motor Klp98A mediates apical to basal Wg transport".

Reviewer 2 Advance Summary and Potential Significance to Field:
This paper identifies and describes the function of Klp98, a kinesin-3 family member, in the polarized endosomal trafficking of Wg in Drosophila epithelium.

Reviewer 2 Comments for the Author:
In this revised version, the authors have taken into consideration my comments and modified the text and the focus of their Manuscript. I congratulate them for this work and I believe that in its present form the article can be considered for publication at Development. I only have minor suggestions for improvement.

1- To specifically address my first concern, the authors have developed and included an assay to live-visualize apical and basal Wg secretion in wing imaginal discs. These new data are presented in Figure 7. For Figure 7C, I recommend the authors to specify the number of independent experiments
they have conducted and to draw on the image the region of interest considered for quantitation, as it is hard to appreciate what a ROI of 150x150 represents (and also because on panel C, part of the basal section is black, apparently out of focus). Together with the data on Rab4-positive compartment, it provides evidence that Wg can be recycled apically. These data support the conclusion that 'Klp98A-independent Wg recycling is sufficient for signal propagation'. They have amended the title and the text of their manuscript accordingly.

We thank the reviewer for this evaluation. We changed the respective figure legend to specifically state the number of WID dissected and experiments conducted. We have furthermore included an exemplary depiction of the 150x150 ROI used for image quantification.

2- In the discussion section, they have now explained in detail the analogy between Klp98A and Kif16B functions. They hey also provided a reviewer figure illustrating the fact that microtubule organization is not impacted by Klp98A silencing. Nonetheless, at this magnification, the distribution of MT differs in anterior versus posterior compartment. It would have been a good idea to show control discs for comparison. Also, it would have been interesting to show MT +ends (using EB1) at the stage analyzed for Wg secretion.

We agree with the reviewer that a more detailed investigation of MT organization during larval wing development would be interesting in the context of Wg secretion. We thank the reviewer for the nice suggestion to track MT plus ends using EB1 and have indeed recently acquired an EB1-GFP overexpression fly strain that we plan to use for further studies.

Reviewer 3 Advance Summary and Potential Significance to Field: See previous round of reviews.

Reviewer 3 Comments for the Author:
The authors have successfully addressed the majority of my points. However, there is one remaining major point (4) that hasn't been addressed by the authors and should be addressed instead. If the author address this point, I believe the paper can be published in Development. In my point 4), I asked the authors to increase the sample size to demonstrate the validity of their negative conclusions. The authors however responded with 3 arguments:

1) "As the wing imaginal disc is a well characterized model system for morphogen trafficking research, we performed experiments in several crosses and dissected 6-10 different imaginal discs for our conclusions".
I disagree with this statement. Proper statistical tests are specifically designed to define what is the minimum number of discs needed to reach a given conclusion. In addition, dissecting more than 6 to 10 discs is a task requiring about two weeks of work.

2) "With these replicates we are able to observe significant differences in Klp98A knockdown in

© 2020. Published by The Company of Biologists under the terms of the Creative Commons Attribution License (https://creativecommons.org/licenses/by/4.0/).

respect to Wg localization and endosomal trafficking. However, other markers are not significantly different."
This is not a logically valid point.

3) "we have included mean values and 95% confidence intervals in Supplementary Table 2 for a clearer data representation"
This is another way of presenting the same data, which has no relation to whether the sample size is appropriate or not. In addition, the table is not precise (fore example one average is out of the 95% confidence interval), and in some cases, as expected, the 95% confidence intervals are very large.

We agree with reviewer 3 that in order to conclude true equivalence from non-significant data, true negative and false negative results need to be carefully distinguished. Generally, three different approaches can be chosen in order to statistically show the absence of an effect: (1) power analysis,
(2) equivalence testing and (3) confidence intervals.

For statistical a priori power analysis, an effect size needs to be defined that sets a threshold between biologically meaningful and negligible results. Deciding about such a threshold in a complex in vivo context is a difficult task and can depend on a researcher's interpretation of which effect strength should be considered relevant. A priori power analysis for an assumed effect size (Cohens d) of 0.5 (corresponding to an "intermediate" effect size) and a desired power of 0.8, demands a sample size of 64 WID per group. This is highly unusual in cell-biological research and not used in comparable publications in the field. It furthermore does not appear to be resourceful, especially in the current times. Generally, as of today, quantifications are rarely performed in *Drosophila* morphogen publications, certainly even less with power analysis.

In our previously revised manuscript version 1, we have - for the above-mentioned reasons - chosen not to perform power analysis and have instead included confidence intervals. Based on confidence intervals, the interpretation of a result as within or out of a biologically meaningful range can be made independently by each reader.

As an alternative approach to further validate negative data, equivalence testing can be performed. With this traditional hypothesis testing approach, the null hypothesis is defined in a way that the difference between groups is expected to cross a certain threshold in order to be practically relevant. Similarly to power analysis, a (subjective) threshold needs to be set to discriminate meaningful from negligible effects. According to recent publications, interference with Wg secretion or signal transfer can be expected to lead to strong effects on target activation. In one recent publication that includes quantitative image analysis, interference with Wg signal transfer reduced activation of the target distalless by 40-75% (Chaudhary and Boutros, Plos Genetics 2019). In the case of senseless, typically observed effects even reflect an on/off state (effect strength of 100%) (Chaudhary and Boutros, Plos Genetics 2019). Based on these data we assume that at least a 25% difference on target activation could be expected if a meaningful biological effect was to occur upon Klp98A knockdown. Hence, we have performed equivalence testing using the rather conservative threshold of 0.25 on our already existing data for senseless, distalless and vestigial activation (Figure 6). We were able to confirm with 95% confidence that any theoretically possible effect on target activation does not exceed this threshold.

Hence, we believe that we have provided enough evidence to conclude (beyond reasonable doubt) that there is no meaningful effect of Klp98A knockdown on Wg signal transduction. In line with that, there is no wing phenotype of Klp98A knockdown. To clarify, we now include a corresponding sentence in line 288 "With comparable stainings and sample sizes, other genes involved in Wnt signaling such as Sec6 (Chaudhary and Boutros, 2019) previously showed an effect on Wnt target gene expression and mention the p-values, equivalence testing as well as a corrected table with all 95% CI intervals in our results, figure legends and methods section. Changes to the mansucript are highlighted in green.

We hope that our changes are now sufficient to meet your statistical expectations and you do not deem an increase of our sample size to n=64 per group (adding up to 128 per staining, or 384

© 2020. Published by The Company of Biologists under the terms of the Creative Commons Attribution License (https://creativecommons.org/licenses/by/4.0/).

WID for Figure 6G alone) as absolutely required for the publication of our manuscript.

We hope that our improvements make our manuscript now suitable for publication in Development.

Thank you for your understanding.

Third decision letter

MS ID#: DEVELOP/2019/186833

MS TITLE: Kinesin motor Klp98A mediates apical to basal Wg transport

AUTHORS: Leonie Witte, Karen Linnemannstoens, Kevin Schmidt, Mona Honemann-Capito, Ferdinand Grawe, Andreas Wodarz, and Julia Christina Gross
ARTICLE TYPE: Research Article

I have read carefully the response to the reviewers, in particular your response with regards to statistics and in light of this I am happy to tell you that your manuscript has been accepted for publication in Development, pending our standard ethics checks.

© 2020. Published by The Company of Biologists under the terms of the Creative Commons Attribution License (https://creativecommons.org/licenses/by/4.0/).
